# Design, Synthesis and Preliminary Biological Evaluation of Novel Benzyl Sulfoxide 2-Indolinone Derivatives as Anticancer Agents

**DOI:** 10.3390/molecules22111979

**Published:** 2017-11-16

**Authors:** Lin Tang, Tao Peng, Gang Wang, Xiaoxue Wen, Yunbo Sun, Shouguo Zhang, Shuchen Liu, Lin Wang

**Affiliations:** Beijing Institute of Radiation Medicine, Beijing 100850, China; adamtl@126.com (L.T.); peng-tao@sohu.com (T.P.); wgang85@126.com (G.W.); crystalcat@vip.sina.com (X.W.); sunyunbo0919@126.com (Y.S.)

**Keywords:** sulfoxide, 2-indolinone derivatives, tyrosine kinase inhibitor, anticancer

## Abstract

In this work, a series of novel benzyl sulfoxide 2-indolinone derivatives was designed and synthesized as potent anticancer agents. Tyrosine kinase inhibitory activity assay indicated that most of the compounds showed significant activity. The in vitro antiproliferative activity of these compounds was further investigated against five human cancer cell lines (HeLa, HepG2, MCF-7, SCC-15, and A549). Several compounds exhibited evident activities. Among them, (*Z*)-3-(((4-bromobenzyl)sulfinyl)methylene)indolin-2-one (**6j**) and (*Z*)-3-((benzylsulfinyl)methylene)-5-bromoindolin-2-one (**6o**) were found to be effective tyrosine kinase inhibitors (IC_50_ = 1.34 and 2.69 μM, respectively) in addition to having noteworthy antitumor potential (the average IC_50_ value of **6j** or **6o** was less than 40 μM). This class of novel derivatives has promising potential for further development as anticancer agents.

## 1. Introduction

Malignant tumors threaten human health and life [1]. Conventional treatments for cancer, including surgery, chemotherapy, and radiation, have significantly improved the prognosis and survival of cancer patients. However, several disadvantages, such as severe surgical trauma and severe damage to normal tissue cells, have stunted their therapeutic uses. Therefore, development of more specific drugs than existing ones is becoming a hot research topic supported by many governments [2,3,4].

Targeted therapy is a new approach for cancer treatment. It has some advantages, such as specificity, and few side effects, which traditional methods do not have. For targeted cancer therapies, small-molecule inhibitors of tyrosine kinase activity are ideal candidates that target tumor cells via binding to intracellular molecules [5,6,7,8,9]. In recent years, 28 small-molecule kinase inhibitors, which have been actively pursued as promising targeted therapeutics, have been approved by the US Food and Drug Administration (FDA) [10,11].

**Figure 1:**
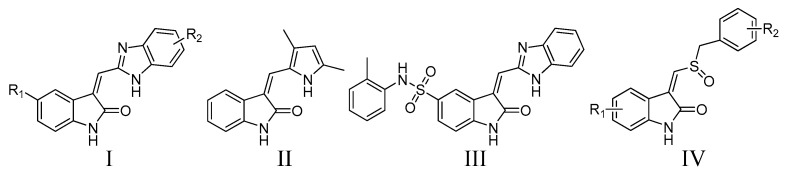
Chemical structures of **I**, **II**, **III** and **IV**.

2-Indolone, also known as oxindole, is a kind of aromatic heterocyclic compound. 2-Indolone structures can be found in many natural alkaloids. For instance, indirubin, an effective component of antileukemic drugs isolated from the traditional Chinese medicine qingdai (indigo naturalis), is a bisindole antitumor drug that can inhibit the growth of multiple transplanted animal tumors [12]. After years of research, a large number of synthesized 2-indolinone derivatives have been found to show widespread biological activities, such as anti-inflammatory, antimicrobial, and antitumor effects [13,14]. Semaxanib (**SU5416**, II in Figure 1), first reported in 1996, is a tyrosine kinase inhibitor drug designed by Sugen as a cancer therapeutic. Semaxanib is a potent and selective synthetic inhibitor of the Flk-1/KDR vascular endothelial growth factor (VEGF) receptor tyrosine kinase. It targets the VEGF pathway, and both in vivo and in vitro studies have demonstrated its antiangiogenic potential [15]. A related compound, SU11248 was further developed by Sugen and then by Pfizer and was FDA approved as sunitinib (Sutent) for the treatment of renal carcinoma in January 2006 [16]. Moreover, many other tyrosine kinase inhibitors, such as PHA-665752, SU11274, TSU-68 (orantinib), and BIBF-1000, were developed as antitumor drugs [17,18,19]. The majority of compounds with tyrosine kinase inhibitory activity are 3-methylene-substituted indolinones or substituted at C-3 with Schiff base structures [15,16,17,18,19,20,21,22,23].

Recently, our laboratory reported a new class of 2-indolinone derivatives (I in Figure 1) based on structure features derived from **SU5416** (II in Figure 1). This new class of 2-indolinone derivatives exhibited excellent tyrosine kinaseinhibitory activity and anticancer activity. Among them, **Indo 5** (III in Figure 1) was the most active compound that exhibited excellent biological activities both in vitro and in vivo [24]. Moreover, **Indo 5** has become a preclinical anticancer drug candidate in our institution. To overcome the disadvantage of **Indo 5** and obtain more effective and ideal antitumor agents, another series of novel compounds (IV in Figure 1) bearing 3-methylene-substituted indolinones were designed based on these findings. The basic tricyclic structure (including the structure of 2-indolinone and a benzene ring) was reserved because at least three rings are considered necessary for the anticancer activity. The sulfoxide group was introduced to this parent nucleus structure for the first time, due to its broad-spectrum biological activities, such as insecticidal, fungicidal, and antitumor effects.

## 2. Results and Discussion

### 2.1. Chemistry

**Scheme 1:**
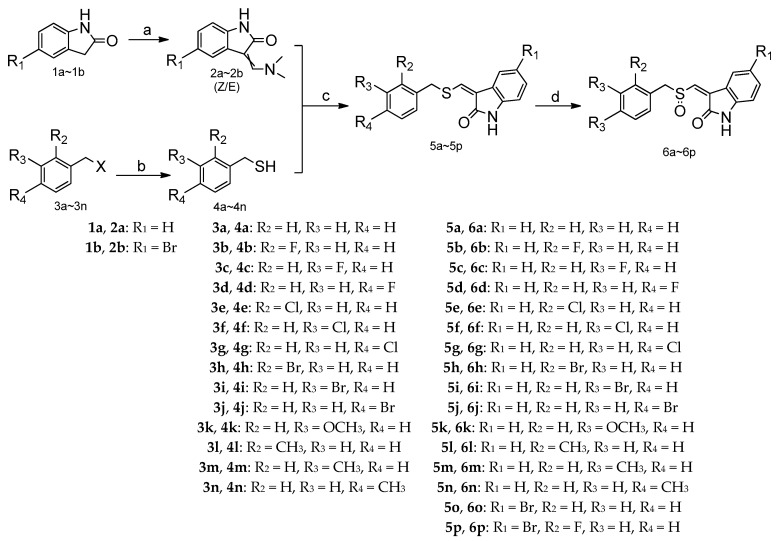
Reagents and conditions: (a) DMF-DMA, toluene, room temperature, 2~4 h; (b) (i) thiourea, ethanol, reflux, 10 min, (ii) sodium hydroxide solution, reflux, 1 h; (c) TEA, methanol, room temperature, 12 h; (d) H_2_O_2_, acetic acid, 40 °C, 4~5 h.

The target compounds were synthesized via a four-step synthetic route from indolin-2-ones (**1**) as shown in Scheme 1. Compounds **2a**–**2b** were synthesized from indolin-2-ones and DMA-DMF as described in the literature [25]. Thiourea was employed to convert benzylic halides (**3a**–**3n**) to the corresponding benzylic thiols (**4a**–**4n**) in a one-pot reaction via the basic hydrolysis of isothiouronium salt [26]. Compounds **5a**–**2p** were synthesized from the reaction of **2a**–**2b** with **4a**–**4o** in the presence of TEA [25]. Finally, the sulfides **5a**–**5p** were oxidized to obtain the corresponding sulfoxides (**6a**–**6p**) with H_2_O_2_ in acetic acid [27]. All the synthesized compounds were purified using recrystallization or silica gel column chromatography. The structures of the target compounds were characterized using ^1^H-NMR, ^13^C-NMR, and HRMS spectral analyses.

### 2.2. Biological Activity

The 16 designed compounds were first evaluated for tyrosine kinase inhibitory activity using ELISA assay. **Indo 5** was used as a reference compound. The kit was self-made and is a very effective and economical method for preliminary tyrosine kinase inhibitory activity evaluation [28]. The IC_50_ values for each compound are summarized in Table 1. Apparently, most of the compounds showed significant tyrosine kinase inhibitory activity. The IC_50_ values of 15 compounds (**6a**, **6b**, **6g**–**6k**, **6m**–**6o**) were much lower than that of **Indo 5**. Compound **6j** exhibited the most potent activity with an IC_50_ value of 1.34 μM.

To investigate the relationship between anticancer activity and tyrosine kinase inhibitory activity, these compounds were further evaluated for in vitro cytotoxicity against five cancer cell lines (human cervical carcinoma cell line HeLa, human breast carcinoma cell line MCF-7, human liver carcinoma cell line HepG2, human tongue carcinoma cell line SCC-15, and human lung carcinoma A549) using MTS assay [29]. **Indo 5** was also used as a reference compound. The IC_50_ values for each compound are summarized in Table 2.

In the cytotoxic assay, 10 compounds (**6a**–**6d**, **6j**, **6k** and **6m**–**6p**) showed potent antineoplastic activity against the HeLa cell line. In particular, compounds **6a**, **6b** and **6o** exhibited stronger activity than **Indo 5**. Four compounds (**6a**, **6c**, **6j** and **6o**) showed good antitumor activity against the MCF-7 cell line. Three compounds (**6d**, **6j** and **6o**) and two compounds (**6j** and **6o**) exhibited considerable cytotoxic activity against the HepG2 cell line and the SCC-15 cell line, respectively. Nevertheless, almost all compounds did not exhibit significant anticancer activity against the A549 cell line. Notably, the more effective compounds **6j** and **6o** exhibited comparable antiproliferative activity against four cancer cell lines compared with compound **Indo 5**. In general, most of the compounds with good tyrosine kinase inhibitory activity also showed significant anticancer activity. **6j** and **6o** were two of the most potent compounds. However, **6l** and **6p** exhibited both weak tyrosine inhibitory activity and anticancer activity. This finding implied a significant correlation between tyrosine inhibitory activity and anticancer activity.

A structure activity relationship study revealed that the introduction of methyl and halogen groups at the 2,3,4-positions of the benzene ring has no evident effect on activity, whereas an increase in activity was observed with para-bromo substitution. The presence of bromo at the 5-position of the indole ring also enhanced activity to a great extent. Unfortunately, the biological activities were simultaneously decreased due to the poor aqueous solubility of this class of sulfoxide derivatives.

## 3. Experimental Section

### 3.1. Chemistry

All chemicals and reagents were purchased from commercial suppliers, of reagent grade, and were used without further purification. Melting points were recorded in an open capillary tube and were uncorrected. Reactions were monitored using TLC and performed on silica gel glass plates containing 60 GF-254. Visualization was achieved by UV light (λ_max_ = 254 or 365 nm). Purification of compounds was performed using silica gel (200–300 mesh) column chromatography. ^1^H-NMR and ^13^C-NMR spectra were recorded using Bruker (Palo Alto, CA, USA) AV-400 and AV-600 NMR spectrometers in DMSO-*d*_6_ with TMS as internal standard. Mass spectral data were obtained using electron spray ionization on a Micromass ZabSpec high-resolution mass spectrometer (Karlsruhe, Germany).

Note: Only the synthesis and characterization of target compounds are presented in this article. The intermediates mentioned in Scheme 1 are described in Supplementary Materials.

#### General Procedure for the Synthesis of Compounds (**6a**–**6p**)

To an ice cold solution of **5a**–**5p** (1.0 mmol, 1 eq.) in 50 mL acetic acid, 30% H_2_O_2_ (1.2 mmol, 1.2 eq.) was added. Then, the reaction was heated to 40 °C for approximately 4–5 h. After completion of the reaction (monitored using TLC), the mixture was poured into ice water. The formed yellow precipitate was filtered, washed with water, and dried under vacuum. The crude product was mixed with 20 mL of anhydrous ethyl acetate and stirred for 2 h at room temperature. The solid was filtered, washed with anhydrous ethyl acetate and dried to obtaint the target compounds (**6a**–**6p**).

*(Z)-3-((Benzylsulfinyl)methylene)indolin-2-one* (**6a**). Obtained in 31.1% yield, yellow solid. m.p. 191–192 °C. ^1^H-NMR (400 MHz, DMSO-*d*_6_) *δ* (ppm): 4.20 (d, 1H, *J* = 12.6 Hz), 4.32 (d, 1H, *J* = 12.6 Hz), 6.88 (d, 1H, *J* = 7.9 Hz), 7.01 (t, 1H, *J* = 8.0 Hz), 7.30–7.41 (m, 6H), 7.60 (s, 1H), 7.72 (d, 1H, *J* = 7.6 Hz), 10.86 (s, 1H, NH); ^13^C-NMR (150 MHz, DMSO-*d*_6_) *δ* (ppm): 166.649, 145.054, 143.953, 132.652, 131.857, 130.754, 128.768, 128.314, 123.012, 122.210, 121.056, 110.707, 60.339; HRMS-ESI (*m*/*z*) Calcd. for C_16_H_14_NO_2_S [M + H]^+^: 284.0745, Found: 284.0738.

*(Z)-3-(((2-Fluorobenzyl)sulfinyl)methylene)indolin-2-one* (**6b**). Obtained in 23.6% yield, yellow solid. m.p. 183–184 °C. ^1^H-NMR (400 MHz, DMSO-*d*_6_) *δ* (ppm): 4.30 (d, 1H, *J* = 12.9 Hz), 4.37 (d, 1H, *J* = 12.9 Hz), 6.83 (d, 1H, *J* = 7.8 Hz), 6.96 (t, 1H, *J* = 7.6 Hz), 7.14–7.40 (m, 5H), 7.47 (s, 1H), 7.66 (d, 1H, *J* = 7.6 Hz), 10.80 (s, 1H, NH); ^13^C-NMR (150 MHz, DMSO-*d*_6_) *δ* (ppm): 166.580, 161.998, 160.392, 144.414, 143.975, 133.266, 133.245, 133.083, 131.936, 130.620, 130.567, 124.651, 124.633, 123.054, 122.222, 121.024, 118.763, 118.662, 115.711, 115.567, 110.689, 52.692; HRMS-ESI (*m*/*z*) Calcd. for C_16_H_13_FNO_2_S [M + H]^+^: 302.0651, Found: 302.0645.

*(Z)-3-(((3-Fluorobenzyl)sulfinyl)methylene)indolin-2-one* (**6c**). Obtained in 39.1% yield, yellow solid. m.p. 186–187 °C. ^1^H-NMR (400 MHz, DMSO-*d*_6_) *δ* (ppm): 4.25 (d, 1H, *J* = 12.8 Hz), 4.36 (d, 1H, *J* = 12.5 Hz), 6.88 (d, 1H, *J* = 7.6 Hz), 7.01 (t, 1H, *J* = 7.6 Hz), 7.16–7.45 (m, 5H), 7.56 (s, 1H), 7.72 (d, 1H, *J* = 7.4 Hz), 10.87 (s, 1H, NH); ^13^C-NMR (150 MHz, DMSO-*d*_6_) *δ* (ppm): 166.775, 166.595, 163.148, 161.529, 161.438, 144.057, 143.941, 143.468, 138.142, 135.404, 134.226, 134.175, 133.327, 132.602, 132.530, 132.277, 130.868, 130.813, 130.783, 127.409, 127.226, 126.967, 123.089, 122.565, 122.437, 120.871, 119.687, 117.735, 117.590, 117.497, 117.355, 115.560, 115.456. 115.422, 115.319, 110.967, 110.935, 59.690, 57.929; HRMS-ESI (*m*/*z*) Calcd. for C_16_H_13_FNO_2_S [M + H]^+^: 302.0651, Found: 302.0646.

*(Z)-3-(((4-Fluorobenzyl)sulfinyl)methylene)indolin-2-one* (**6d**). Obtained in 25.7% yield, yellow solid. m.p. 193–194 °C. ^1^H-NMR (400 MHz, DMSO-*d*_6_) *δ* (ppm): 4.21 (d, 1H, *J* = 12.6 Hz), 4.32 (d, 1H, *J* = 12.9 Hz), 6.88 (d, 1H, *J* = 7.8 Hz), 7.00 (t, 1H, *J* = 7.6 Hz), 7.19–7.38 (m, 5H), 7.54 (s, 1H), 7.71 (d, 1H, *J* = 7.6 Hz), 10.86 (s, 1H, NH); ^13^C-NMR (150 MHz, DMSO-*d*_6_) *δ* (ppm): 166.235, 165.996, 162.940, 162.828, 161.207, 144.377, 143.747, 143.589, 138.367, 134.651, 132.623, 132.566, 132.390, 132.333, 132.153, 132.100, 131.845, 131.475, 130.926, 127.609, 127.081, 126.113, 122.640, 121.812, 121.703, 120.638, 119.415, 115.430, 115.385, 115.226, 115.124, 114.147, 114.007, 110.313, 58.850, 57.098, 56.608; HRMS-ESI (*m*/*z*) Calcd. for C_16_H_13_FNO_2_S [M + H]^+^: 302.0651, Found: 302.0645.

*(Z)-3-(((2-Chlorobenzyl)sulfinyl)methylene)indolin-2-one* (**6e**). Obtained in 31.6% yield, yellow solid. m.p. 202–203 °C. ^1^H-NMR (400 MHz , DMSO-*d*_6_) *δ* (ppm): 4.43 (d, 1H, *J* = 12.6 Hz), 4.54 (d, 1H, *J* = 12.6 Hz), 6.85 (d, 1H, *J* = 7.8 Hz), 7.00 (t, 1H, *J* = 7.6 Hz), 7.28–7.48 (m, 5H), 7.55 (s, 1H), 7.69 (d, 1H, *J* = 7.8 Hz), 10.79 (s, 1H, NH); ^13^C-NMR (150 MHz, DMSO-*d*_6_) *δ* (ppm): 166.113, 143.561, 143.526, 133.967, 133.080, 132.910, 131.566, 129.803, 129.346, 129.000, 126.969, 122.680, 121.802, 120.632, 110.258, 56.057; HRMS-ESI (*m*/*z*) Calcd. for C_16_H_13_ClNO_2_S [M + H]^+^: 318.0356, Found: 318.0350.

*(Z)-3-(((3-Chlorobenzyl)sulfinyl)methylene)indolin-2-one* (**6f**). Obtained in 28.6% yield, yellow solid. m.p. 192–194 °C. ^1^H-NMR (400 MHz, DMSO-*d*_6_) *δ* (ppm): 4.24 (d, 1H, *J* = 12.6 Hz), 4.35 (d, 1H, *J* = 12.3 Hz), 6.88 (d, 1H, *J* = 7.8 Hz), 7.01 (t, 1H, *J* = 7.6 Hz), 7.28–7.42 (m, 5H), 7.56 (s, 1H), 7.72 (d, 1H, *J* = 7.3 Hz), 10.87 (s, 1H, NH); ^13^C-NMR (150 MHz, DMSO-*d*_6_) *δ* (ppm): 166.239, 144.172, 143.636, 133.870, 132.849, 132.550, 131.526, 130.155, 130.084, 129.048, 127.848, 122.680, 121.826, 120.600, 110.323, 59.132; HRMS-ESI (*m*/*z*) Calcd. for C_16_H_13_ClNO_2_S [M + H]^+^: 318.0356, Found: 318.0349.

*(Z)-3-(((4-Chlorobenzyl)sulfinyl)methylene)indolin-2-one* (**6g**). Obtained in 23.8% yield, yellow solid. m.p. 193–195 °C. ^1^H-NMR (400 MHz, DMSO-*d*_6_) *δ* (ppm): 4.22 (d, 1H, *J* = 12.9 Hz), 4.34 (d, 1H, *J* = 12.6 Hz), 6.88 (d, 1H, *J* = 7.6 Hz), 7.01 (t, 1H, *J* = 7.6 Hz), 7.30–7.46 (m, 5H), 7.52 (s, 1H), 7.71 (d, 1H, *J* = 7.3 Hz), 10.84 (s, 1H, NH); ^13^C-NMR (150 MHz, DMSO-*d*_6_) *δ* (ppm): 166.235, 144.302, 143.609, 132.789, 132.463, 132.177, 131.495, 130.376, 128.309, 122.684, 121.818, 120.620, 110.311, 58.852; HRMS-ESI (*m*/*z*) Calcd. for C_16_H_13_ClNO_2_S [M + H]^+^: 318.0356, Found: 318.0349.

*(Z)-3-(((2-Bromobenzyl)sulfinyl)methylene)indolin-2-one* (**6h**). Obtained in 50.0% yield, yellow solid. m.p. 198–199 °C. ^1^H-NMR (400 MHz, DMSO-*d*_6_) *δ* (ppm): 4.46 (d, 1H, *J* = 12.9 Hz), 4.56 (d, 1H, *J* = 12.9 Hz), 6.86 (d, 1H, *J* = 7.8 Hz), 7.00 (t, 1H, *J* = 7.1 Hz), 7.25–7.49 (m, 4H), 7.56 (s, 1H), 7.64 (d, 1H, *J* = 7.8 Hz), 7.70 (d, 1H, *J* = 7.6 Hz), 10.78 (s, 1H, NH); ^13^C-NMR (150 MHz, DMSO-*d*_6_) *δ* (ppm): 166.083, 143.529, 143.271, 133.056, 132.659, 131.582, 130.720, 129.989, 127.520, 124.781, 122.701, 121.794, 120.638, 110.256, 58.383; HRMS-ESI (*m*/*z*) Calcd. for C_16_H_13_BrNO_2_S [M + H]^+^: 363.9830, Found: 363.9825.

*(Z)-3-(((3-Bromobenzyl)sulfinyl)methylene)indolin-2-one* (**6i**). Obtained in 51.7% yield, yellow solid. m.p. 193–194 °C. ^1^H-NMR (400 MHz, DMSO-*d*_6_) *δ* (ppm): 4.25 (d, 1H, *J* = 12.6 Hz), 4.27 (d, 1H, *J* = 12.6 Hz), 6.90 (d, 1H, *J* = 7.9 Hz), 7.03 (t, 1H, *J* = 7.7 Hz), 7.32–7.58 (m, 6H), 7.74 (d, 1H, *J* = 7.3 Hz), 10.89 (s, 1H, NH); ^13^C-NMR (150 MHz, DMSO-*d*_6_) *δ* (ppm): 166.239, 144.176, 143.637, 134.135, 132.955, 132.548, 131.524, 130.722, 130.437, 129.414, 122.678, 121.824, 121.440, 120.595, 110.321, 59.105; HRMS-ESI (*m*/*z*) Calcd. for C_16_H_13_BrNO_2_S [M + H]^+^: 363.9830, Found: 363.9826.

*(Z)-3-(((4-Bromobenzyl)sulfinyl)methylene)indolin-2-one* (**6j**). Obtained in 40.0% yield, yellow solid. m.p. 195–196 °C. ^1^H-NMR (400 MHz, DMSO-*d*_6_) *δ* (ppm): 4.21 (d, 1H, *J* = 12.6 Hz), 4.33 (d, 1H, *J* = 12.6 Hz), 6.88 (d, 1H, *J* = 7.7 Hz), 7.01 (t, 1H, *J* = 7.6 Hz), 7.27–7.60 (m, 6H), 7.72 (d, 1H, *J* = 7.6 Hz), 10.87 (s, 1H, NH); ^13^C-NMR (150 MHz, DMSO-*d*_6_) *δ* (ppm): 166.237, 144.304, 143.616, 132.641, 132.507, 132.465, 131.493, 131.234, 130.779, 122.690, 121.816, 121.401, 120.618, 110.313, 58.905; HRMS-ESI (*m*/*z*) Calcd. for C_16_H_13_BrNO_2_S [M + H]^+^: 363.9830, Found: 363.9824.

*(Z)-3-(((3-Methoxybenzyl)sulfinyl)methylene)indolin-2-one* (**6k**). Obtained in 32.1% yield, yellow solid. m.p. 181–182 °C. ^1^H-NMR (400 MHz, DMSO-*d*_6_) *δ* (ppm): 3.73 (s, 3H,), 4.17 (d, 1H, *J* = 12.6 Hz), 4.29 (d, 1H, *J* = 12.6 Hz), 6.87–6.93 (m, 4H), 7.01 (t, 1H, *J* = 7.6 Hz), 7.27–7.33 (m, 2H), 7.60 (s, 1H,), 7.72 (d, 1H, *J* = 7.3 Hz), 10.85 (s, 1H, NH); ^13^C-NMR (150 MHz, DMSO-*d*_6_) *δ* (ppm): 166.247, 159.128, 144.589, 143.539, 132.843, 132.270, 131.451, 129.394, 122.597, 121.802, 120.668, 115.837, 113.495, 110.299, 60.004, 54.952; HRMS-ESI (*m*/*z*) Calcd. for C_17_H_16_NO_3_S [M + H]^+^: 314.0851, Found: 314.0845.

*(Z)-3-(((2-Methylbenzyl)sulfinyl)methylene)indolin-2-one* (**6l**). Obtained in 32.4% yield, yellow solid. m.p. 198–199 °C. ^1^H-NMR (400 MHz, DMSO-*d*_6_) *δ* (ppm): 2.42 (s, 3H,), 4.28 (d, 1H, *J* = 12.6 Hz), 4.35 (d, 1H, *J* = 12.9 Hz), 6.88 (d, 1H, *J* = 7.6 Hz), 7.02 (t, 1H, *J* = 7.4 Hz), 7.18–7.34 (m, 5H), 7.72–7.75 (m, 2H), 10.80 (s, 1H, NH); ^13^C-NMR (150 MHz, DMSO-*d*_6_) *δ* (ppm): 166.223, 144.925, 143.470, 137.920, 132.198, 131.673, 131.467, 130.167, 129.833, 128.093, 125.763, 122.614, 121.816, 120.676, 110.293, 57.551; HRMS-ESI (*m*/*z*) Calcd. for C_17_H_16_NO_2_S [M + H]^+^: 298.0892, Found: 298.0894.

*(Z)-3-(((3-Methylbenzyl)sulfinyl)methylene)indolin-2-one* (**6m**). Obtained in 52.1% yield, yellow solid. m.p. 174–175 °C. ^1^H-NMR (400 MHz, DMSO-*d*_6_) *δ* (ppm): 2.32 (s, 3H,), 4.17 (d, 1H, *J* = 12.6 Hz), 4.29 (d, 1H, *J* = 12.6 Hz), 6.91 (d, 1H, *J* = 7.8 Hz), 7.04 (t, 1H, *J* = 7.6 Hz), 7.15–7.34 (m, 5H), 7.64 (s, 1H), 7.75 (d, 1H, *J* = 7.6 Hz), 10.87 (s, 1H, NH); ^13^C-NMR (150 MHz, DMSO-*d*_6_) *δ* (ppm): 166.255, 144.804, 143.535, 137.485, 132.200, 131.430, 131.388, 130.906, 128.568, 128.291, 127.429, 122.587, 121.806, 120.666, 110.201, 60.095; HRMS-ESI (*m*/*z*) Calcd. for C_17_H_16_NO_2_S [M + H]^+^: 298.0892, Found: 298.0894.

*(Z)-3-(((4-Methylbenzyl)sulfinyl)methylene)indolin-2-one* (**6n**). Obtained in 32.8% yield, yellow solid. m.p. 186–188 °C. ^1^H-NMR (400 MHz, DMSO-*d*_6_) *δ* (ppm): 2.31 (s, 3H,), 4.15 (d, 1H, *J* = 12.6 Hz), 4.27 (d, 1H, *J* = 12.6 Hz), 6.88 (d, 1H, *J* = 7.8 Hz), 7.01 (t, 1H, *J* = 6.7 Hz), 7.17–7.33 (m, 5H), 7.58 (s, 1H), 7.72 (d, 1H, *J* = 7.6 Hz), 10.86 (s, 1H, NH); ^13^C-NMR (150 MHz, DMSO-*d*_6_) *δ* (ppm): 166.249, 144.798, 143.524, 137.219, 132.171, 131.412, 130.252, 128.951, 128.321, 122.591, 121.796, 120.676, 110.293, 59.638; HRMS-ESI (*m*/*z*) Calcd. for C_17_H_16_NO_2_S [M + H]^+^: 298.0892, Found: 298.0894.

*(Z)-3-((Benzylsulfinyl)methylene)-5-bromoindolin-2-one* (**6o**). Obtained in 30.8% yield, yellow solid. m.p. 200–201 °C. ^1^H-NMR (400 MHz, DMSO-*d*_6_) *δ* (ppm): 4.21 (d, 1H, *J* = 12.6 Hz), 4.33 (d, 1H, *J* = 12.6 Hz), 6.88 (d, 1H, *J* = 7.9 Hz), 7.01 (t, 1H, *J* = 7.6 Hz), 7.27–7.34 (m, 3H), 7.53 (s, 1H), 7.59 (d, 1H, *J* = 8.4 Hz), 7.72 (d, 1H, *J* = 7.6 Hz), 10.87 (s, 1H, NH); ^13^C-NMR (150 MHz, DMSO-*d*_6_) *δ* (ppm): 166.237, 144.300, 143.618, 132.507, 132.467, 131.493,131.234, 130.779, 122.688, 121.816, 121.401, 120.618, 110.315, 58.905; HRMS-ESI (*m*/*z*) Calcd. for C_16_H_13_BrNO_2_S [M + H]^+^: 363.9830, Found: 363.9826.

*(Z)-5-Bromo-3-(((2-fluorobenzyl)sulfinyl)methylene)indolin-2-one* (**6p**). Obtained in 22.2% yield, yellow solid. m.p. 208–209 °C. ^1^H-NMR (400 MHz, DMSO-*d*_6_) *δ* (ppm): 4.34 (d, 1H, *J* = 12.9 Hz), 4.41 (d, 1H, *J* = 12.9 Hz), 6.83 (d, 1H, *J* = 8.2 Hz), 7.19–7.49 (m, 5H), 7.71 (s, 1H), 8.00 (s, 1H), 10.97 (s, 1H, NH); ^13^C-NMR (150 MHz, DMSO-*d*_6_) *δ* (ppm): 165.798, 161.598, 159.960, 146.583, 142.634, 133.716, 132.874, 131.554, 130.273, 120.220, 125.429, 124.261, 122.688, 118.274, 118.173, 115.331, 115.189, 113.602, 112.212, 52.321; HRMS-ESI (*m*/*z*) Calcd. for C_16_H_12_BrFNO_2_S [M + H]^+^: 381.9736, Found: 381.9729.

### 3.2. Biological Evaluation

#### 3.2.1. Tyrosine Kinase Inhibitory Activity Assay

The tyrosine kinase inhibitory activity assay was performed as described in the literature [28]. Details of the experimental procedures are presented in Supplementary Materials associated with this article. The IC_50_ was calculated using GraphPad Prism Software (GraphPad Prism 5, La Jolla, CA, USA). For each concentration, at least three wells were performed to calculate the average parameter.

#### 3.2.2. Cytotoxic Activity Assays

##### Cell Culture

The five types of human cancer cell lines (HeLa, HepG2, MCF-7, SCC-15, and A549) were cultured aseptically using Dulbecco’s modified Eagle’s medium (DMEM) supplemented with 10% (*v*/*v*) fetal bovine serum (FBS) and penicillin (100 units mL^−1^)/streptomycin (100 mg mL^−1^), at pH 7.2 and 5% CO_2_ humidified atmosphere at 37 °C. After attaining 80% confluence, the cells were trypsinized with 0.25 trypsin–EDTA and diluted with media to a fixed number of cells.

##### MTS Assay

Cytotoxic activity was assessed using the standard MTS method by using triplicate assay. The cells were seeded into 96-well plates containing the medium at the density of 4000–6000 cells/mL (100 μL/well). The compounds were dissolved in DMSO to the concentration of 100 mM and diluted in a culture medium to the concentrations needed. After 24 h, the cultured cells were treated with concentrations of synthesized compounds (3.125 μM to 100 μM for tumor cells) for 48 h. After 48 h of incubation, the supernatant was replaced by fresh medium (100 μL/well) and 10 μL MTS reagent ([3-(4,5-dimethylthiazol-2-yl)-5-(3-carboxymethoxyphenyl)-2-(4-sulfophenyl)-2*H*-tetrazolium, inner salt]) was added to each well. The plate was further incubated for 3 h at 37 °C in 5% CO2. The optical absorbance in individual wells was determined at 492 nm using a microplate reader. The inhibition rates were calculated by the following formula:

Inhibition rate (%) = (OD_negative control_ − OD_sample_)/(OD_negative control_ − OD_blank_) × 100%

IC_50_ was calculated using GraphPad Prism Software (GraphPad Prism 5, La Jolla, CA, USA). For each concentration, at least three wells were performed to calculate the average parameter.

## 4. Conclusions

In summary, a series of novel benzyl sulfoxide 2-indolinone derivatives was synthesized and evaluated in vitro for tyrosine kinase inhibitory activity and potential cytotoxic activity against five types of cancer cell lines. Most of the compounds under investigation exhibited significant tyrosine kinase inhibitory activity. Compounds **6j** and **6o** not only showed significant tyrosine kinase inhibitory activity but also exhibited obvious antiproliferative activity against four tested cancer cell lines. Several compounds showed encouraging activity in some cases with interesting selectivity against the HeLa cell line relative to the other four cancer cell lines. The findings highlighted the potential of this class of derivatives as new anticancer agents. Further detailed research will be conducted to evaluate the molecular mechanism underlying the anticancer activity of these compounds. In addition, hydrophilic groups will be introduced to the parent nucleus structure to improve the aqueous solubility of the compounds to enhance their anti-tumor activity.

## Figures and Tables

**Table 1:** The tyrosine kinase inhibitory activity of compounds **6a**~**6p**.

Compound	Tyrosine Kinase Inhibitory Activity
	IC_50_ (μM)
**6a**	3.16 ± 0.31
**6b**	3.74 ± 0.19
**6c**	9.32 ± 0.66
**6d**	9.30 ± 0.47
**6e**	5.09 ± 0.12
**6f**	5.96 ± 0.14
**6g**	3.30 ± 0.03
**6h**	3.52 ± 0.15
**6i**	3.32 ± 0.19
**6j**	1.34 ± 0.03
**6k**	2.23 ± 0.14
**6l**	16.02 ± 0.05
**6m**	3.85 ± 0.09
**6n**	4.39 ± 0.23
**6o**	2.69 ± 0.29
**6p**	7.93 ± 0.20
**Indo 5**	11.60 ± 0.79

**Table 2:** The anti-cancer activity of compounds **6a**~**6p**.

Compound	HeLa	MCF-7	HepG2	SCC-15	A549
	IC_50_ (μM)	IC_50_ (μM)	IC_50_ (μM)	IC_50_ (μM)	IC_50_ (μM)
**6a**	11.17 ± 0.42	35.18 ± 1.16	52.08 ± 0.94	58.85 ± 0.75	>100
**6b**	13.99 ± 0.27	49.12 ± 1.08	47.82 ± 1.47	>100	>100
**6c**	24.37 ± 0.54	31.54 ± 0.82	42.02 ± 0.70	49.87 ± 0.93	>100
**6d**	25.02 ± 0.68	49.12 ± 0.29	36.25 ± 0.53	64.97 ± 1.47	90.28 ± 2.14
**6e**	70.88 ± 1.17	>100	96.83 ± 0.95	>100	>100
**6f**	60.27 ± 1.98	93.02 ± 1.50	86.50 ± 1.15	96.78 ± 3.76	>100
**6g**	40.92 ± 0.88	92.63 ± 0.97	75.35 ± 0.72	>100	>100
**6h**	79.87 ± 1.57	>100	98.29 ± 2.04	>100	>100
**6i**	48.42 ± 0.14	75.06 ± 1.93	71.77 ± 2.18	82.14 ± 3.34	64.91 ± 1.57
**6j**	24.05 ± 0.52	30.77 ± 0.29	36.90 ± 0.49	34.90 ± 0.34	44.01 ± 1.89
**6k**	22.67 ± 0.64	59.08 ± 037	49.36 ± 1.33	86.72 ± 5.16	62.34 ± 2.00
**6l**	>100	81.57 ± 2.62	>100	>100	>100
**6m**	27.03 ± 1.20	41.43 ± 2.20	36.04 ± 2.13	50.33 ± 1.51	79.32 ± 0.79
**6n**	27.76 ± 0.78	55.09 ± 1.51	58.99 ± 0.97	90.49 ± 2.32	59.56 ± 0.97
**6o**	18.22 ± 0.66	32.34 ± 0.77	54.44 ± 0.88	28.63 ± 1.58	54.863 ± 0.66
**6p**	19.83 ± 0.25	76.42 ± 1.50	>100	>100	>100
**Indo 5**	19.64 ± 0.46	24.19 ± 0.49	22.15 ± 0.41	28.16 ± 1.08	29.16 ± 0.29

## Supplementary Materials

Supplementary materials are available online.
