# Supplementary material for: Design, Synthesis and Preliminary Biological Evaluation of Novel Benzyl Sulfoxide 2-Indolinone Derivatives as Anticancer Agents
Source: Molecules. 2017 Nov 16;22(11):1979. doi: 10.3390/molecules22111979 (PMC6150246; doi:10.3390/molecules22111979)

Design, Synthesis and preliminary biological evaluation of novel benzyl sulfoxide 2-indolinone derivatives as anti-cancer agents

Lin Tang, Tao Peng, Gang Wang, Xiaoxue Wen, Yunbo Sun, Shouguo Zhang*, Shuchen Liu*, Lin Wang *

Beijing Institute of Radiation Medicine, Beijing 100850, P. R. China

*corresponding author. Tel.: +86 010 66932239

E-mail addresses: wanglin@bmi.ac.cn & wanglin07@sina.com

Supplementary Materials

1. Synthesis of Intermediates

1.1. *5-Bromo-oxindole* (1b)

Oxindole (2.50g, 19mmol, 1eq.) was dissolved in 80mL of boiling water. Bromine (3 g, 19mmol, 1eq.) and potassium bromide (4.50g, 38mmol, 2eq.) were dissolved in 10mL of water and then added dropwise to a warm, stired solution of oxindole in 5 min. A white prepcipitate appeared. The mixture was stored for 20min in refrigerator. The precipitate was filtered, extensively washed with water and dried in vacuum at elevated temperature to give 2.70g of 5-bromo-oxindole as a colorless solid.

Obtained in 67.8% yield, colorless solid. M.p. 221-223 ℃. ^{1}H-NMR(400Hz, DMSO-d_6) δ (ppm)：3.47(s, 2H), 6.72(d, 1H, *J*=8.4Hz), 7.29-7.34(m, 2H), 10.46(s, 1H, NH). It was identified by comparison with its ^{1}H-NMR spectra with the literature data.[1]

1.2. General procedure for the synthesis of compounds (2a~2b)

DMF-DMA (10.64mL, 80mmol, 5eq.) was added to a solution of the appropriate indole **(2a~2b)** (16 mmol, 1eq.) in toluene (30mL). The mixture was stirred for 2-4h, The precipitate was collected by filtration and washed with dichloromethane to give **2a~2b**.

(Z/E)-3-(N,N-dimethylaminomethylene)indolin-2-one (**2a**). Obtained in 58.4% yield, beige solid. Z/E mixture 1/1. M.p. 208-209 ℃. ^{1}H-NMR(400Hz, DMSO-d_6) δ (ppm)：3.26(s br, 3H), 3.29(s br, 6H), 3.62 (s br, 3H), 6.69(d, 1H, *J*=8.0Hz), 6.75-6.85(m, 4H), 6.90(t, 1H, *J*=8.0Hz), 7.25(d, 1H, *J*=8.0Hz), 7.37-7.40(m, 2H), 7.53 (s, 1H), 9.92(s br, 1H, NH) , 10.00(s br, 1H, NH). It was identified by comparison with its ^{1}H-NMR spectra with the literature data.[2]

(Z/E)-5-bromo-3-(N,N-dimethylaminomethylene)indolin-2-one (**2b**). Obtained in 78.2% yield, beige solid. Z/E mixture 3/1. M.p. 230-232 ℃. Z-isomer- ^{1}H-NMR(400Hz, DMSO-d_6) δ (ppm)：3.26(s br, 3H), 3.65 (s br, 3H), 6.62(d, 1H, J=8.0Hz), 6.94(dd, 1H, J_1=8.0Hz, J_2=2.0Hz), 7.48(s, 1H), 7.67(s, 1H), 10.08(s, 1H, NH). It was identified by comparison with its ^{1}H-NMR spectra with the literature data.[3]

1.3. General procedure for the synthesis of compounds (4a~4n)

To a warm solution of thiourea(0.92g, 12.07mmol, 1.2eq.) in 30 mL of ethanol was added benzylic halides (**3a~3n**) (10mmol, 1eq.). The reaction mixture was heated to reflux for 10 min (monitored by TLC). The resulting colorless transparent solution was concentrated in vacuo almost to dryness and then a solution of NaOH(1.2g, 30mmol) in 10mL water was added (a white precipitate was formed). The reaction mixture was heated to reflux for an hour and cooled (a precipitate shouldn't appear; if it does , the mixture should be heated longer). The reaction mixture was extracted with CH_2Cl_2 (100 mL). The organic phase was dried over Na_2SO_4 and concentrated to give the corresponding benzylic thiols (**4a~4n**) as a colorless liquid.

Benzyl mercaptane (**4a**). Benzyl thiol was purchased from commercial suppliers was of reagents grade and used without further purification.

2-fluorobenzyl mercaptane (**4b**). Obtained in 59.0% yield, colorless liquid. It was identified by comparison with its physical data with the literature data. [4]

3-fluorobenzyl mercaptane (**4c**). Obtained in 64.0% yield, colorless liquid. It was identified by comparison with its physical data with the literature data. [5]

4-fluorobenzyl mercaptane (**4d**). Obtained in 83.5% yield, colorless liquid. It was identified by comparison with its physical data with the literature data. [6]

2-chlorobenzyl mercaptane (**4e**). Obtained in 100% yield, colorless liquid. It was identified by comparison with its physical data with the literature data. [7]

3-chlorobenzyl mercaptane (**4f**). Obtained in 90.0% yield, colorless liquid. It was identified by comparison with its physical data with the literature data. [7]

4-chlorobenzyl mercaptane (**4g**). Obtained in 91.0% yield, colorless liquid. It was identified by comparison with its physical data with the literature data. [8]

2-bromobenzyl mercaptane (**4h**). Obtained in 92.0% yield, colorless liquid. It was

identified by comparison with its physical data with the literature data. [9]

3-bromobenzyl mercaptane (**4i**). Obtained in 92.1% yield, colorless liquid. It was identified by comparison with its physical data with the literature data. [7]

4-bromobenzyl mercaptane (**4j**). Obtained in 100% yield, colorless liquid. It was identified by comparison with its physical data with the literature data. [9]

3-methoxybenzyl mercaptane (**4k**). Obtained in 88.8% yield, colorless liquid. It was identified by comparison with its physical data with the literature data. [10]

2-methylbenzyl mercaptan (**4l**). Obtained in 89.5% yield, colorless liquid. It was identified by comparison with its physical data with the literature data. [11]

3-methylbenzyl mercaptan (**4m**). Obtained in 100% yield, colorless liquid. It was identified by comparison with its physical data with the literature data. [12]

4-methylbenzyl mercaptan (**4n**). Obtained in 97.3% yield, colorless liquid. It was identified by comparison with its physical data with the literature data. [10]

1.4. General procedure for the synthesis of compounds (5a~5p)

The mixture of benzylic thiols (**4a~4n**) (10mmol, 1eq.), TEA (18mmol, 1.8eq.) in MeOH (60mL) was stirred for 15 min at 0℃ before addition of 3-(N,N- dimethylaminomethylene) indolin-2-ones **(2a~2b)** (12mmol, 1.2eq.), then was stirred for 12h at RT. After completion of reaction(monitored by TLC), evaporation the methanol, purification by column chromatography.

(Z)-3-((benzylthio)methylene)indolin-2-one **(5a)**. Obtained in 48.7% yield, yellow solid. M.p. 171-173 ℃. ^{1}H-NMR (400MHz , DMSO-*d*$_6$) *δ* (ppm): 4.45(s, 2H), 6.82(d, 1H, *J*=7.8Hz), 6.98(t, 1H, *J*=7.6Hz), 7.18(t, 1H, *J*=7.7Hz), 7.29-7.48(m, 6H), 7.84(s, 1H), 10.42(s, 1H, NH); It was identified by comparison with its ^{1}H-NMR spectra with the literature data.[2]

*(Z/E)3-(((2-fluorobenzyl) thio)methylene)indolin-2-one***(5b).** Obtained in 44.3% yield, yellow solid. Z/E mixture 6/1. M.p. 207-208 ℃. <u>Z-isomer</u>- ^{1}H-NMR (400MHz , DMSO-*d*$_6$) *δ* (ppm): 4.54(s, 2H), 6.83(d, 1H, *J*=8.0Hz), 6.98(t, 1H, *J*=8.0Hz), 7.18(t, 1H, *J*=8.0Hz), 7.28(t, 1H, *J*=8.0Hz), 7.38-7.46(m, 2H), 7.67-7.71(m, 2H), 7.93(s, 1H), 10.45(s, 1H, NH); HRMS-ESI *(m/z)* Calcd. for $C_{16}H_{13}FNOS$ $[M+H]^+$: 286.0696, Found: 286.0687.

(Z/E)-3-(((3-fluorobenzyl) thio)methylene)indolin-2-one **(5c).** Obtained in 31.9% yield, yellow solid. Z/E mixture 9/1. M.p. 159-160 ℃. <u>Z-isomer</u>- ^{1}H-NMR (400MHz , DMSO-*d*$_6$) *δ*

(ppm): 4.47(s, 2H), 6.82(d, 1H, *J*=8.0Hz), 6.98(t, 1H, *J*=8.0Hz), 7.12-7.20(m, 2H), 7.32-7.44(m, 4H), 7.86(s, 1H), 10.45(s, 1H, NH); HRMS-ESI *(m/z)* Calcd. for $C_{16}H_{13}FNOS$ $[M+H]^+$: 286.0696, Found: 286.0687.

(Z/E)-3-(((4-fluorobenzyl) thio)methylene)indolin-2-one **(5d).** Obtained in 45.7% yield, yellow solid. Z/E mixture 6/1. M.p. 138-140 ℃. Z-isomer- ^{1}H-NMR (400MHz , DMSO-d_6) δ (ppm): 4.46(s, 2H), 6.83(d, 1H, *J*=8.0Hz), 6.98(t, 1H, *J*=8.0Hz), 7.16-7.25(m, 3H), 7.40(d, 1H, *J*=8.0Hz), 7.50-7.54(m, 2H), 7.85(s, 1H), 10.44(s, 1H, NH); HRMS-ESI *(m/z)* Calcd. for $C_{16}H_{13}FNOS$ $[M+H]^+$: 286.0696, Found: 286.0687.

(Z/E)-3-(((2-chlorobenzyl) thio)methylene)indolin-2-one **(5e).** Obtained in 51.1% yield, yellow solid. Z/E mixture 6/1. M.p. 138-140 ℃. Z-isomer- ^{1}H-NMR (400MHz , DMSO-d_6) δ (ppm): 4.55(s, 2H), 6.83(d, 1H, *J*=8.0Hz), 6.98(t, 1H, *J*=8.0Hz), 7.18(t, 1H, *J*=8.0Hz), 7.36-7.40(m, 3H), 7.50-7.54(m, 1H), 7.68-7.70(m, 1H), 7.93(s, 1H), 10.44(s, 1H, NH); HRMS-ESI *(m/z)* Calcd. for $C_{16}H_{13}ClNOS$ $[M+H]^+$: 302.0401, Found: 302.0395.

(Z/E)-3-(((3-chlorobenzyl) thio)methylene)indolin-2-one **(5f).** Obtained in 38.2% yield, yellow solid. Z/E mixture 2.5/1. M.p. 145-147 ℃. Z-isomer- ^{1}H-NMR (400MHz , DMSO-d_6) δ (ppm): 4.47(s, 2H), 6.83(d, 1H, *J*=8.0Hz), 6.98(t, 1H, *J*=8.0Hz), 7.18(t, 1H, *J*=8.0Hz), 7.36-7.44(m, 4H), 7.58(s, 1H), 7.86(s, 1H), 10.45(s, 1H, NH); HRMS-ESI *(m/z)* Calcd. for $C_{16}H_{13}ClNOS$ $[M+H]^+$: 302.0401, Found: 302.0395.

(Z/E)-3-(((4-chlorobenzyl) thio)methylene)indolin-2-one **(5g)**. Obtained in 37.8% yield, yellow solid. Z/E mixture 2/1. M.p. 170-173 ℃. Z-isomer- ^{1}H-NMR (400MHz , DMSO-d_6) δ (ppm): 4.46(s, 2H), 6.83(d, 1H, *J*=8.0Hz), 6.98(t, 1H, *J*=8.0Hz), 7.18(t, 1H, *J*=8.0Hz), 7.38-7.51(m, 5H), 7.83(s, 1H), 10.44(s, 1H, NH); HRMS-ESI *(m/z)* Calcd. for $C_{16}H_{13}ClNOS$ $[M+H]^+$: 302.0401, Found: 302.0395.

(Z/E)-3-(((2-bromobenzyl) thio)methylene)indolin-2-one **(5h).** Obtained in 38.2% yield, yellow solid. Z/E mixture 8/1. M.p. 261-262 ℃. Z-isomer- ^{1}H-NMR (400MHz , DMSO-d_6) δ (ppm): 4.54(s, 2H), 6.82(d, 1H, *J*=8.0Hz), 6.98(t, 1H, *J*=8.0Hz), 7.18(t, 1H, *J*=8.0Hz), 7.28(t, 1H, *J*=8.0Hz), 7.37-7.45(m, 2H), 7.67-7.71(m, 2H), 7.93(s, 1H), 10.44(s, 1H, NH); HRMS-ESI *(m/z)* Calcd. for $C_{16}H_{13}BrNOS$ $[M+H]^+$: 345.9896, Found: 345.9885.

(Z/E)-3-(((3-bromobenzyl) thio)methylene)indolin-2-one **(5i).** Obtained in 46.7% yield, yellow solid. Z/E mixture 4/1. M.p. 155-157 ℃. Z-isomer- ^{1}H-NMR (400MHz , DMSO-d_6) δ

(ppm): 4.46(s, 2H), 6.82(d, 1H, *J*=8.0Hz), 6.98(t, 1H, *J*=8.0Hz), 7.18(t, 1H, *J*=8.0Hz), 7.33-7.41(m, 2H), 7.47-7.52(m, 2H), 7.72(s br, 1H), 7.86(s, 1H), 10.45(s, 1H, NH); HRMS-ESI *(m/z)* Calcd. for $C_{16}H_{13}BrNOS$ $[M+H]^+$: 345.9896, Found: 345.9884.

(Z/E)-3-(((4-bromobenzyl) thio)methylene)indolin-2-one **(5j).** Obtained in 24.3% yield, yellow solid. Z/E mixture 10/1. M.p. 186-187 ℃. Z-isomer- ^{1}H-NMR (400MHz , DMSO-d_6) δ (ppm): 4.44(s, 2H), 6.82(d, 1H, *J*=8.0Hz), 6.98(t, 1H, *J*=8.0Hz), 7.18(t, 1H, *J*=8.0Hz), 7.39-7.45(m, 3H), 7.57-7.59(m, 2H), 7.83(s, 1H), 10.43(s, 1H, NH); HRMS-ESI *(m/z)* Calcd. for $C_{16}H_{13}BrNOS$ $[M+H]^+$: 345.9896, Found: 345.9885.

(Z/E)-3-(((3-methoxybenzyl) thio)methylene)indolin-2-one **(5k).** Obtained in 31.3% yield, yellow solid. Z/E mixture 3/1. M.p. 159-162 ℃. Z-isomer- ^{1}H-NMR (400MHz , DMSO-d_6) δ (ppm): 3.76(s, 3H), 4.41(s, 2H), 6.81-7.06(m, 5H), 7.18(t, 1H, *J*=8.0Hz), 7.28(d, 1H, *J*=8.0Hz), 7.42(d, 1H, *J*=8.0Hz), 7.85(s, 1H), 10.43(s, 1H, NH); HRMS-ESI *(m/z)* Calcd. for $C_{17}H_{16}NO_2S$ $[M+H]^+$: 298.0896, Found:298.0887.

(Z/E)-3-(((2-methylbenzyl) thio)methylene)indolin-2-one **(5l).** Obtained in 55.3% yield, yellow solid. Z/E mixture 3/1. M.p. 196-197 ℃. Z-isomer- ^{1}H-NMR (400MHz , DMSO-d_6) δ (ppm): 2.39(s, 3H), 4.47(s, 2H), 6.82(d, 1H, *J*=8.0Hz), 6.97(t, 1H, *J*=8.0Hz), 7.13-7.24(m, 4H), 7.39-7.48(m, 2H), 7.86(s, 1H), 10.42(s, 1H, NH); HRMS-ESI *(m/z)* Calcd. for $C_{17}H_{16}NOS$ $[M+H]^+$: 282.0947, Found:282.0940.

(Z/E)-3-(((3-methylbenzyl) thio)methylene)indolin-2-one **(5m).** Obtained in 47.0% yield, yellow solid. Z/E mixture 13.5/1. M.p. 170-172 ℃. Z-isomer- ^{1}H-NMR (400MHz , DMSO-d_6) δ (ppm): 2.31(s, 3H), 4.41(s, 2H), 6.82(d, 1H, *J*=8.0Hz), 6.98(t, 1H, *J*=8.0Hz), 7.11(dd, 1H, J_1=8.0Hz, J_2=2.0Hz), 7.18(t, 1H, *J*=8.0Hz), 7.24-7.27(m, 3H), 7.40(d, 1H, *J*=8.0Hz), 7.84(s, 1H), 10.43(s, 1H, NH); HRMS-ESI *(m/z)* Calcd. for $C_{17}H_{16}NOS$ $[M+H]^+$: 282.0947, Found:282.0940.

(Z/E)-3-(((4-methylbenzyl) thio)methylene)indolin-2-one **(5n).** Obtained in 47.5% yield, yellow solid. Z/E mixture 10/1. M.p. 168-170 ℃. Z-isomer- ^{1}H-NMR (400MHz , DMSO-d_6) δ (ppm): 2.28(s, 3H), 4.40(s, 2H), 6.82(d, 1H, *J*=8.0Hz), 6.98(t, 1H, *J*=8.0Hz), 7.16-7.20(m, 3H), 7.34(d, 2H, *J*=8.0Hz), 7.39(d, 1H, *J*=8.0Hz), 7.82(s, 1H), 10.42(s, 1H, NH); HRMS-ESI *(m/z)* Calcd. for $C_{17}H_{16}NOS$ $[M+H]^+$: 282.0947, Found:282.0940.

(Z/E)-3-((benzylsulfinyl) thio)-5-bromoindolin-2-one **(5o).** Obtained in 46.7% yield,

yellow solid. Z/E mixture 3/1. M.p. 222-224 ℃. Z-isomer- ^{1}H-NMR (400MHz , DMSO-d_6) δ (ppm): 4.49(s, 2H), 6.79(d, 1H, *J*=8.0Hz), 7.26-7.40(m, 5H), 7.47-7.49(m, 2H), 7.86(s, 1H), 10.56(s, 1H, NH); HRMS-ESI *(m/z)* Calcd. for $C_{16}H_{13}BrNOS$ $[M+H]^+$: 345.9896, Found: 345.9886.

(Z/E)-5-bromo-3-(((2-fluorobenzyl) thio)methylene)indolin-2-one **(5p).** Obtained in 37.3% yield, yellow solid. Z/E mixture 5/1.M.p. 213-214 ℃. Z-isomer- ^{1}H-NMR (400MHz , DMSO-d_6) δ (ppm): 4.54(s, 2H), 6.80(d, 1H, *J*=8.0Hz), 7.23-7.29(m, 2H), 7.35-7.44(m, 3H), 7.61(t, 1H, *J*=8.0Hz), 8.03(s, 1H), 10.61(s, 1H, NH); HRMS-ESI *(m/z)* Calcd. for $C_{16}H_{12}BrFNOS$ $[M+H]^+$: 363.9802, Found: 363.9800.

2. Tyrosine kinase inhibitory activity assay

2.1. Materials and methods

2.1.1. Tyrosine kinase buffer: 10mL lM HEPES(pH7.5), 0.4mL 5% BSA/PBS, 0.2mL 0.1M Na_3V0_4， and 1mL 5M NaCl were added to 88.4mL double distilled water (DDW) (HEPES: Amresco; Na_3V0_4: Sigma).

2.1.2. ATP: Adenosine triphosphate, Amresco.

2.1.3. Extraction of tissue extract PTK: the mouse brain tissue was quickly removed and weighed, followed by addition of five-fold volumes of pre-cooled homogenization buffer for homogenization. After centrifugation at 4℃, 1,000 g for 10 min, nuclei and cell debris were removed. The supernatant S1 was collected and centrifuged at 4℃, 10,000 g for 20 min, and then the supernatant S2 was collected. The precipitate P2, which represents the crude membrane protein fraction, was retained. S2 included cytoplasmic protein, and was used for testing protein tyrosine kinase (PTK) activity. In the detection of the membrane protein fraction, two-fold volumes of lysis buffer was added to P2, and the resultant was placed on ice for 10 min and centrifuged at 4℃, 10,000 g for 10 min. The supernatant S3, which represents the crude membrane protein including soluble membrane protein, was collected for testing PTK activity. Protein content in cytoplasm or membrane protein was detected by using BCA protein concentration kit (purchased from Beyotime Institute of Biotechnology). The tissue extract was stored at -70℃.

2.1.4. Coating of 96-well plate: PTK substrate was dissolved and added in an amount of

100μL to each well. The plate was covered with its lid, and incubated at 4℃ overnight (10-12 hours). Then it was washed with 200μL elution buffer once, and dried at 37℃ for 2 h. After that, it was washed with 10mM PBS once, dried at room temperature, and then stored at 4℃ for further use.

2.1.5. Groups: Blank control: 60μL 1×tyrosine kinase buffer + 50μL 1×tyrosine kinase buffer containing ATP; **Negative control:** 10μL 1×tyrosine kinase buffer + 50μL 1×tyrosine kinase buffer containing tissue extract + 50μL 1×tyrosine kinase buffer containing ATP; **Positive control:** 10μL 1×tyrosine kinase buffer containing L029 + 50μL 1×tyrosine kinase buffer containing tissue extract + 50μL 1×tyrosine kinase buffer containing ATP; **Vehicle control:** 10μL 1×tyrosine kinase buffer containing DMSO + 50μL 1×tyrosine kinase buffer containing tissue extract + 50μL 1×tyrosine kinase buffer containing ATP; **Compounds to be screened**: 10μL 1×tyrosine kinase buffer containing compounds to be tested + 50μL 1× tyrosine kinase buffer containing tissue extract + 50μL 1×tyrosine kinase buffer containing ATP. (First, L029 and the compounds were allowed to interact with the tyrosine kinase tissue extract at room temperature for 10 min in advance, and did the same to the Blank control and the Vehicle control, then 50μL 1×tyrosine kinase buffer containing ATP was added to respective groups)

2.1.6. Tyrosine kinase inhibitory activity assay: 1×tyrosine kinase buffer was prepared by evenly mixing 1 mL 10× tyrosine kinase buffer with 9 mL DDW. The tissue extract was diluted with 1×tyrosine kinase buffer appropriately, mixed with it gently and evenly, and placed on ice. ATP stock solution was mixed with 1×tyrosine kinase buffer at the final concentration of 3mM , and placed on ice. The microplate was added in respective wells with the groups formulated as above, covered with the lid, and incubated at 37℃ for 30 min. The microplate was washed with 200μL elution buffer and tapped until the residual buffer was removed, and this procedure was repeated for five times. To each well, 100μL antibody diluent (antibody elution buffer diluted at a ratio of 1:2000) was added. The microplate was covered with the lid, and incubated at room temperature for 30 min. OPD solution was prepared by addition of 4 mg OPD into a mixture of 4.86 mL 0.1 mol/L citric acid solution and 5.14 mL 0.2 mol/L Na_2HPO_4 solution, followed by addition of 50μL 30% H_20_2 to allow for complete dissolution, and then was protected from light. The antibody solution was

removed. The microplate was washed with 200μL elution buffer and tapped until the residual buffer was removed, and this procedure was repeated for five times. 100μL freshly prepared OPD was added, and reacted at room temperature light tight for precisely 7 min. Negative wells showed an orange color. 100μL 2.5 N H_2SO_4 was added to terminate the reaction. OD was measured at 492 nm.

2.1.7. Sample screening: Samples were screened preliminarily by using triplicate assay. The effect of the samples on tyrosine kinase inhibitory activity was tested, and the inhibition rate was calculated. Inhibition rate (%)=($OD_{negative\ control}$ – OD_{sample}) / ($OD_{negative\ control*}$ – OD_{blank}) × 100% ; *While calculating inhibition rate of positive drugs, $OD_{negative\ control}$ in the equation was replaced with $OD_{vehicle\ control}$ to exclude the influence of DMSO on tyrosine kinase activity.

References

1. You Zi, Zhong-Jian Cai, Shun-Yi Wang, et al. *Org. Lett.* **2014**; 16: 3094 - 3097.

2. S. Praveen Kumar, Jiri Gut, Rita C. Guedes. et al. *Eur. J. Med. Chem.* **2011**; 46: 927-933.

3. Fadoua Bouchikhia, Emilie Rossignola, Martine Sancelmea. et al. *Eur. J. Med. Chem.* **2008**; 43: 2316-2322.

4. Sylvie Mavela, Jean Louis Renoua, Christophe Galtiera. et al. *Arzneimittel-Rorsch.* **2001**; 51: 304-309.

5. Alain Brembilla, Denis Roizard, Jacqueline Schoenleber. et al. *Can. J. Chem.* **1984**, 62: 2330-2336.

6. Fillmore Freeman, Hengyao Lu, Qingbei Zeng. et al. *J. Org. Chem.* **1994**; 59: 4350-4354.

7. Chandra Kant Maurya, Avik Mazumder, Pradeep Kumar Gupta1. *Beilstein J. Org. Chem.* **2017;** 13: 1184-1188.

8. Petra Herzigová, Vera Klimešová, Karel Palát. et al. *Arch. Pnarm.* **2009;** 342: 394-404.

9. Joseph L. Howard, Christiane Schotten, Stephen T. Alstona t. et al. *Chem. Commun.* **2016;** 52: 8448-8451.

10. Tohru Nakamura, Mutsuyoshi Matsumoto. *Synthetic. Commun.* **1999**; 29: 201 - 210.

11. Juan Almena, Francisco Foubelo, Miguel Yus. *J. Org. Chem.* **1996**; 62: 1859-1862.

12. Niklas B. Heine, Armido Studer. *Org. Lett.* **2017**; 19: 4150 - 4153.

3. Copies of ^{1}H-NMR and ^{13}C-NMR for target compounds（6a-6p）

^{1}H-NMR of Compound 6a:

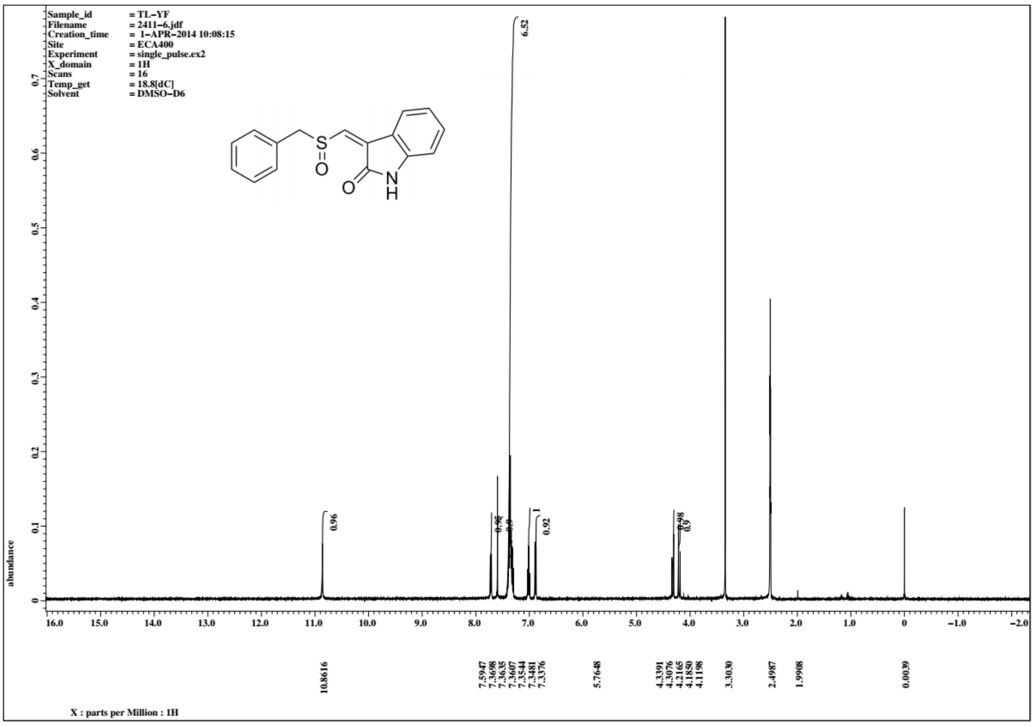

^{1}C-NMR of Compound 6a:

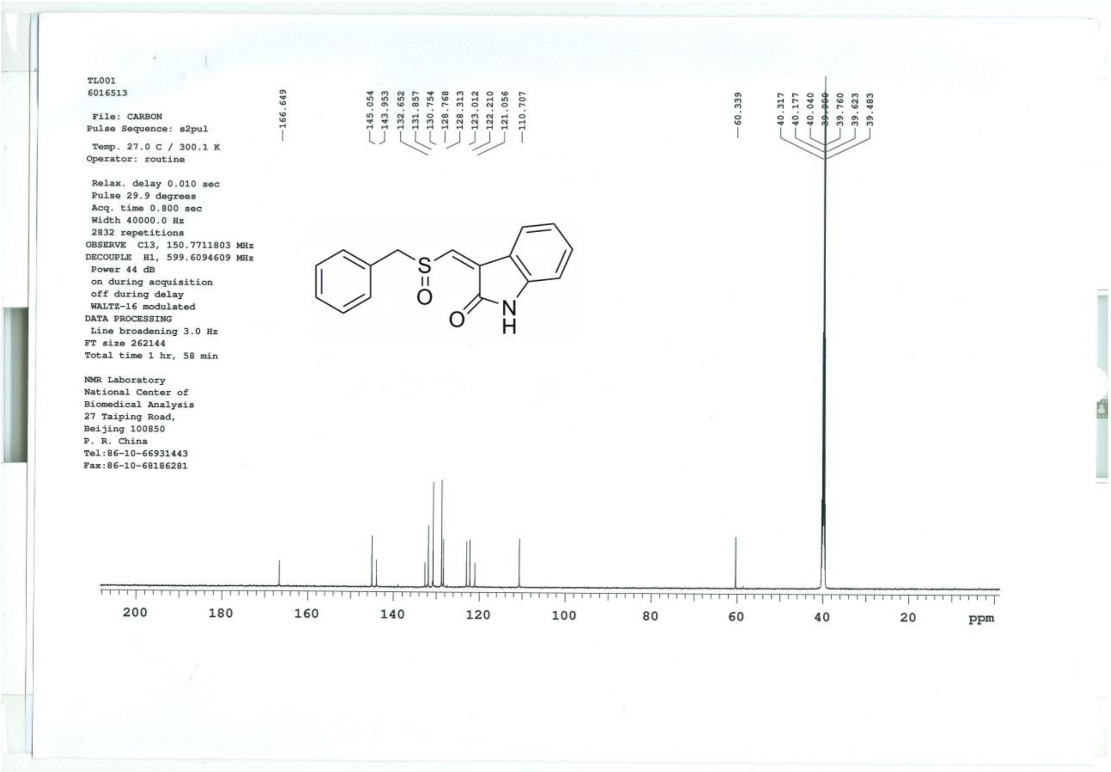

^{1}H-NMR of Compound 6b:

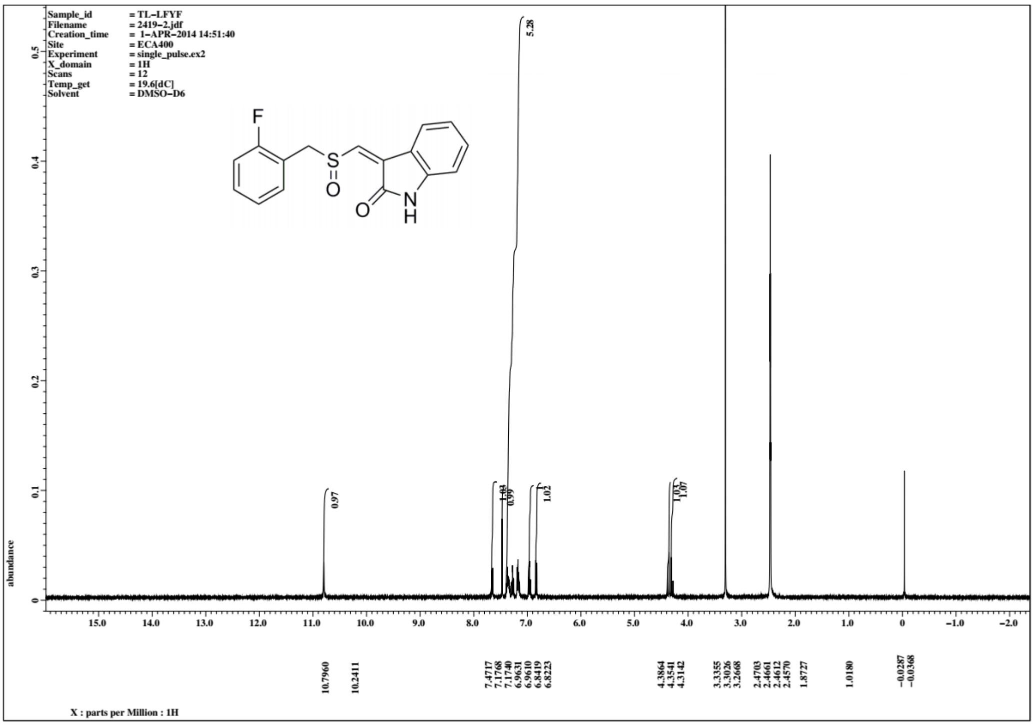

^{1}C-NMR of Compound 6b:

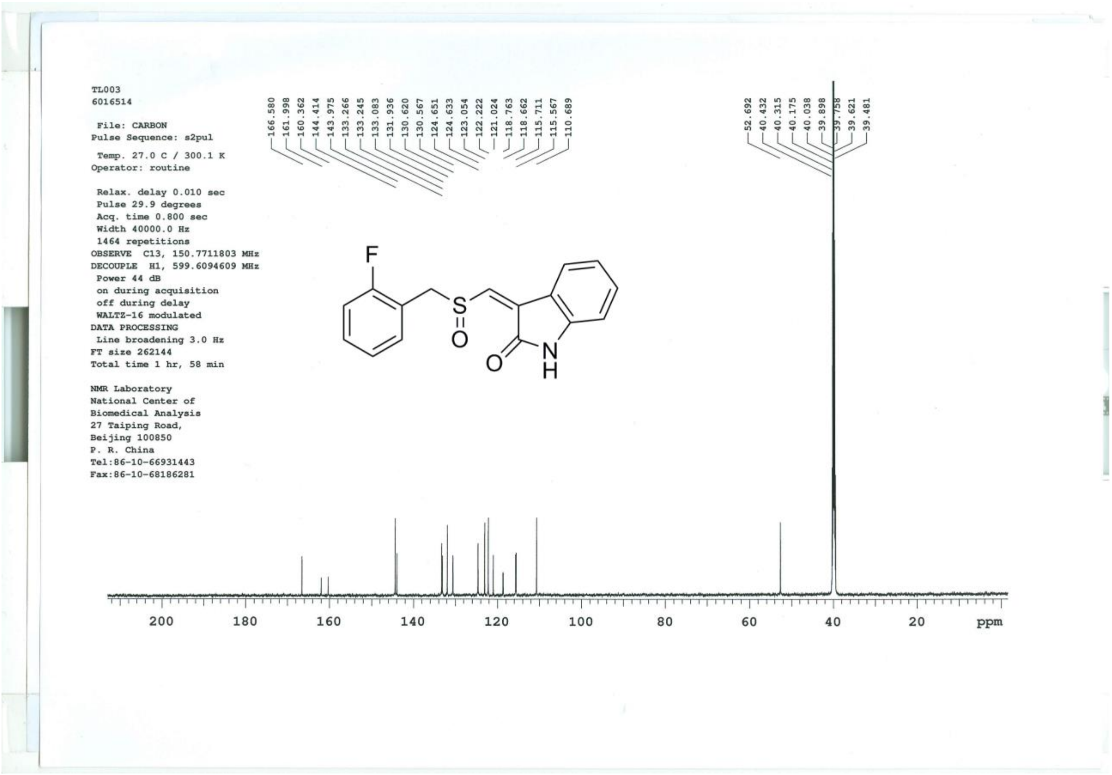

^{1}H-NMR of Compound 6c:

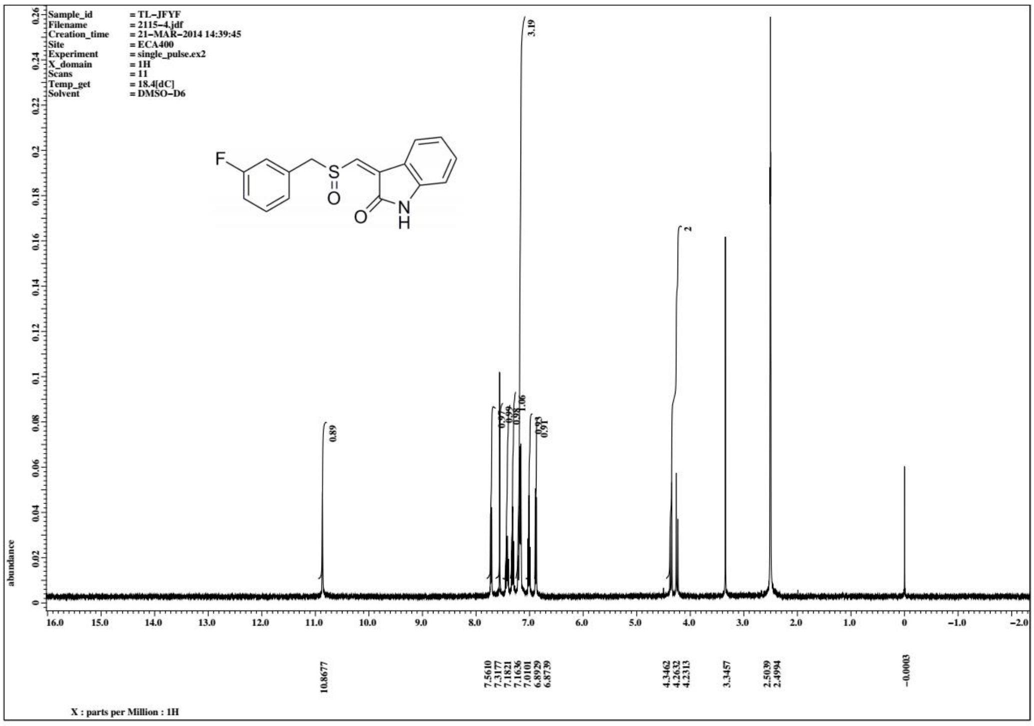

^{1}C-NMR of Compound 6c:

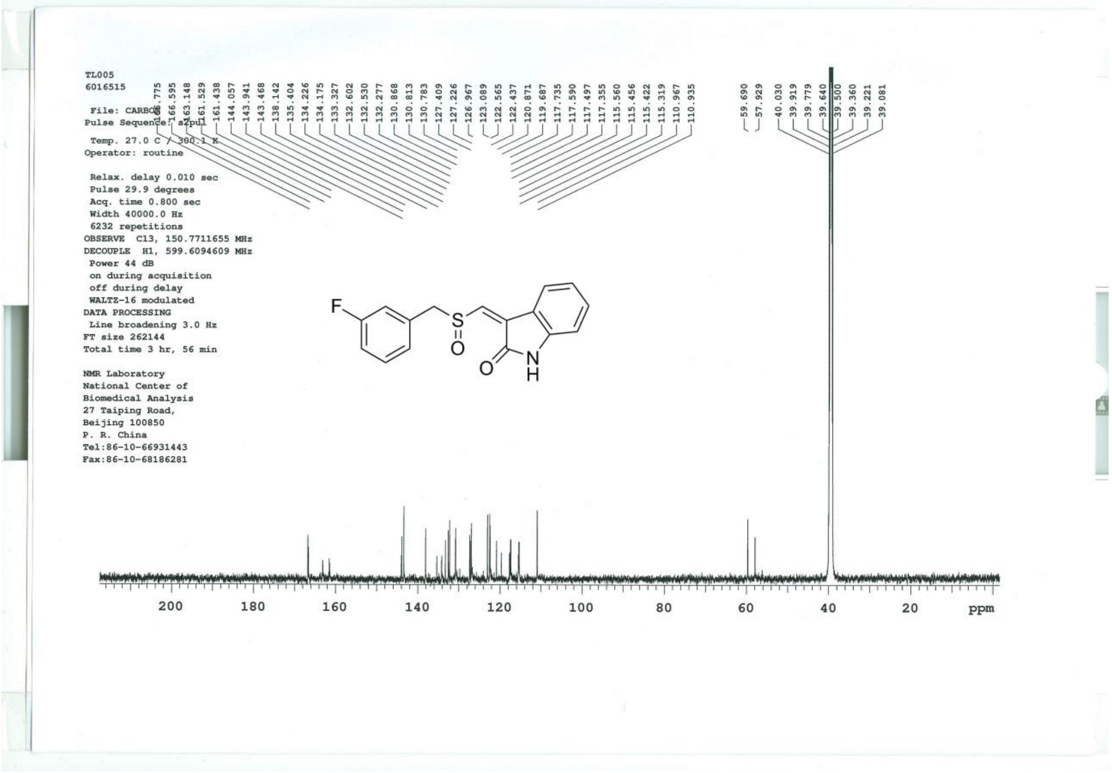

^{1}H-NMR of Compound 6d:

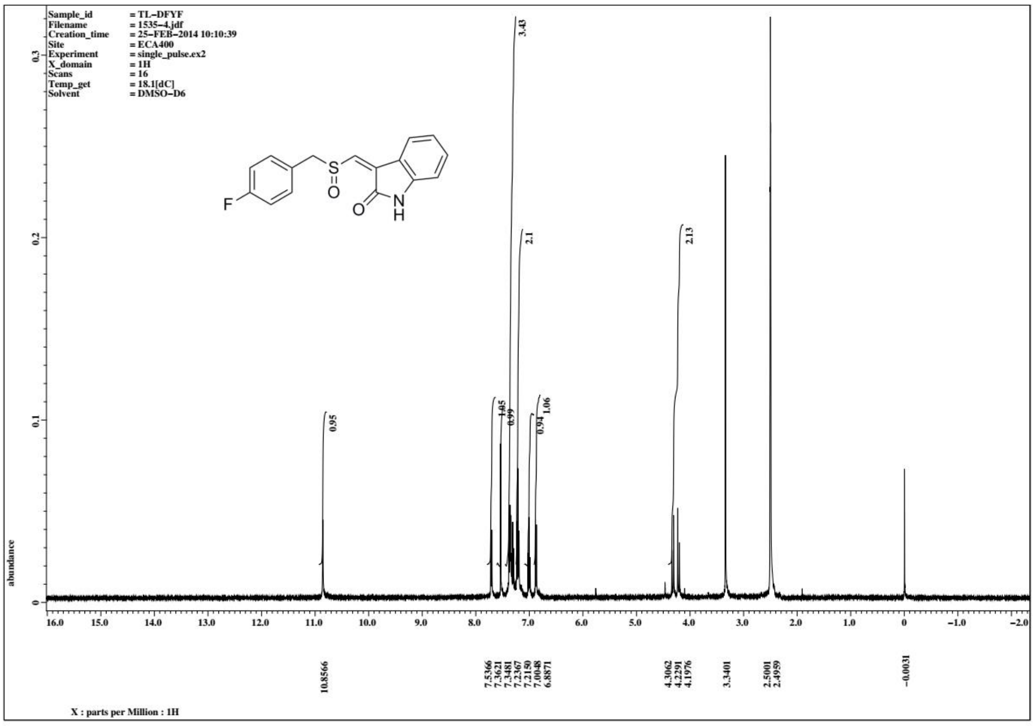

^{1}C-NMR of Compound 6d:

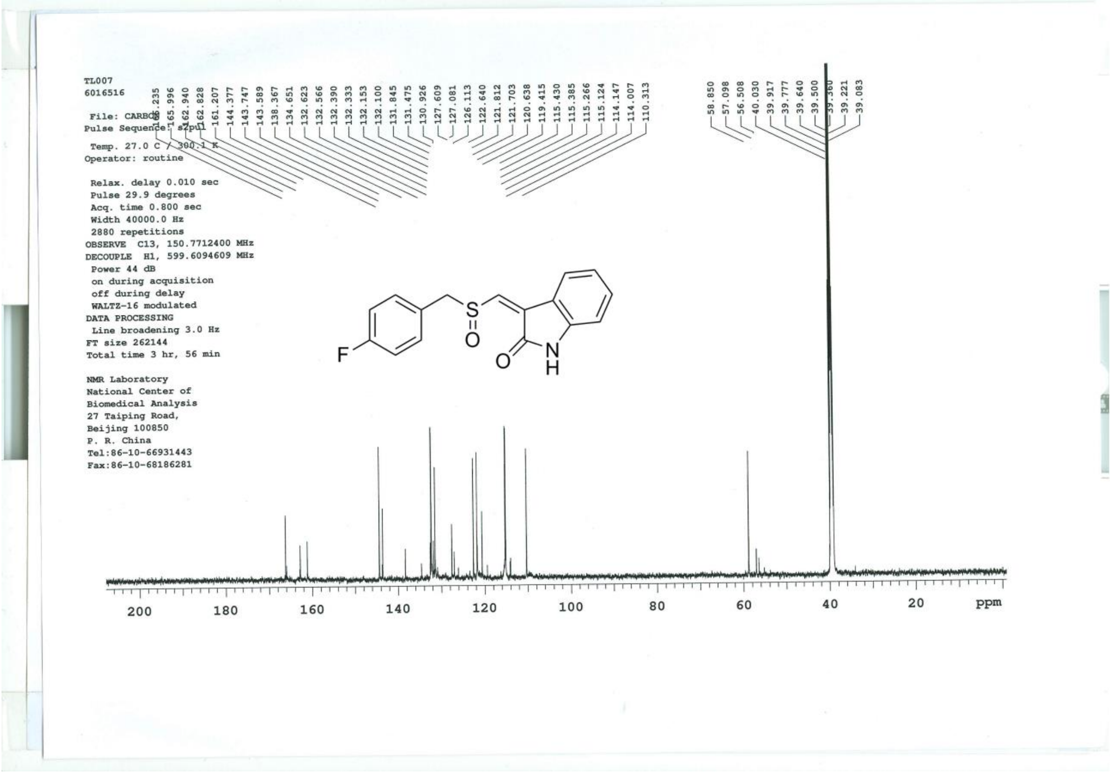

^{1}H-NMR of Compound 6e:

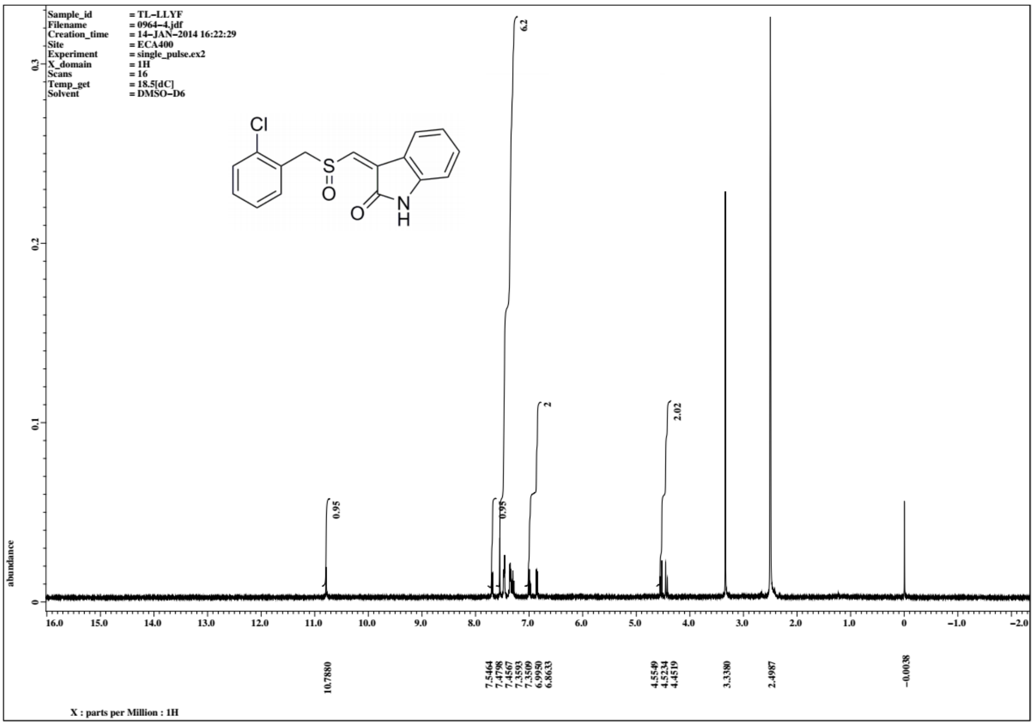

[1]C-NMR of Compound 6e:

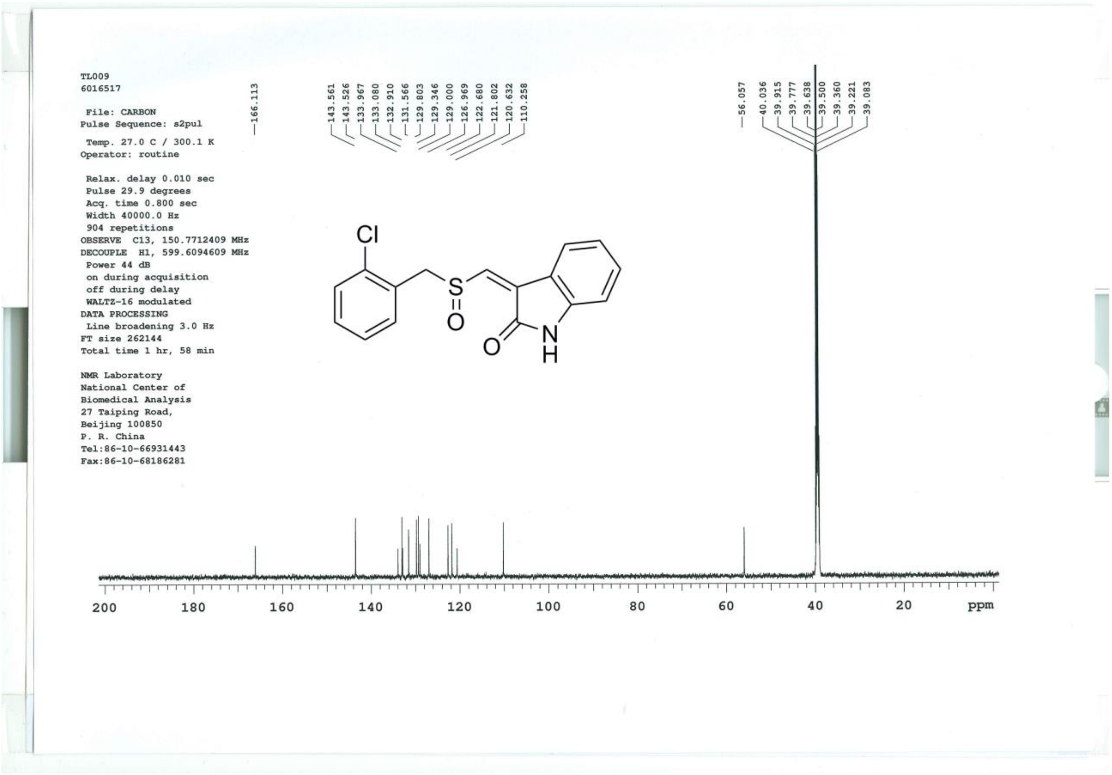

^{1}H-NMR of Compound 6f:

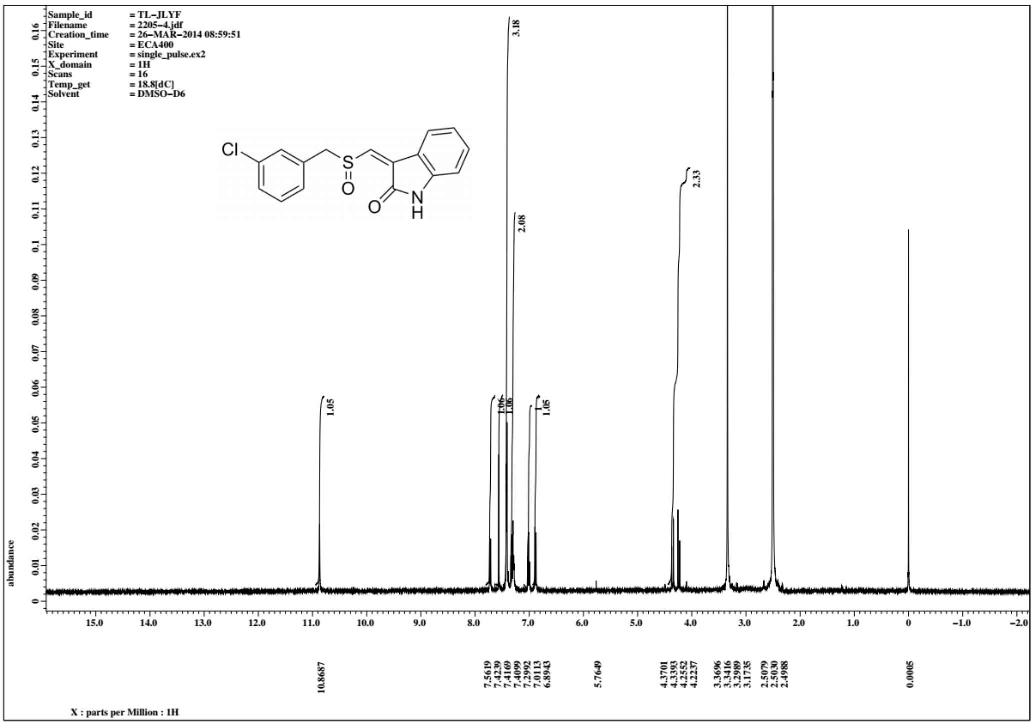

^{1}C-NMR of Compound 6f:

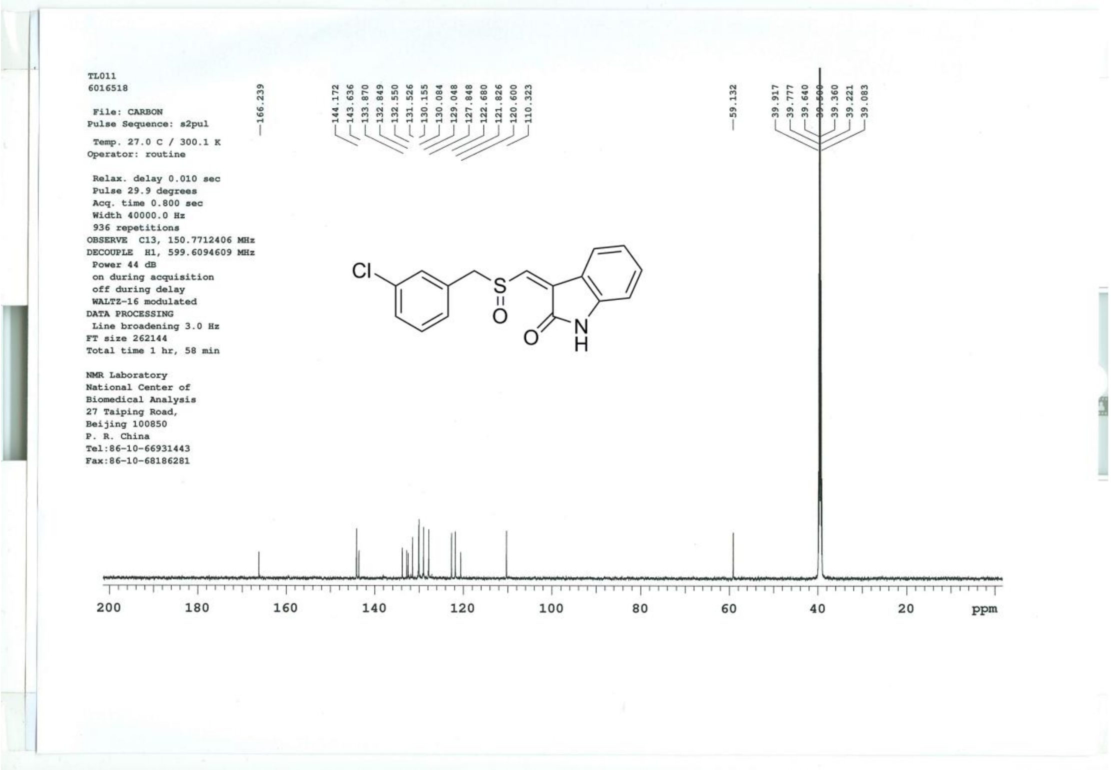

^{1}H-NMR of Compound 6g:

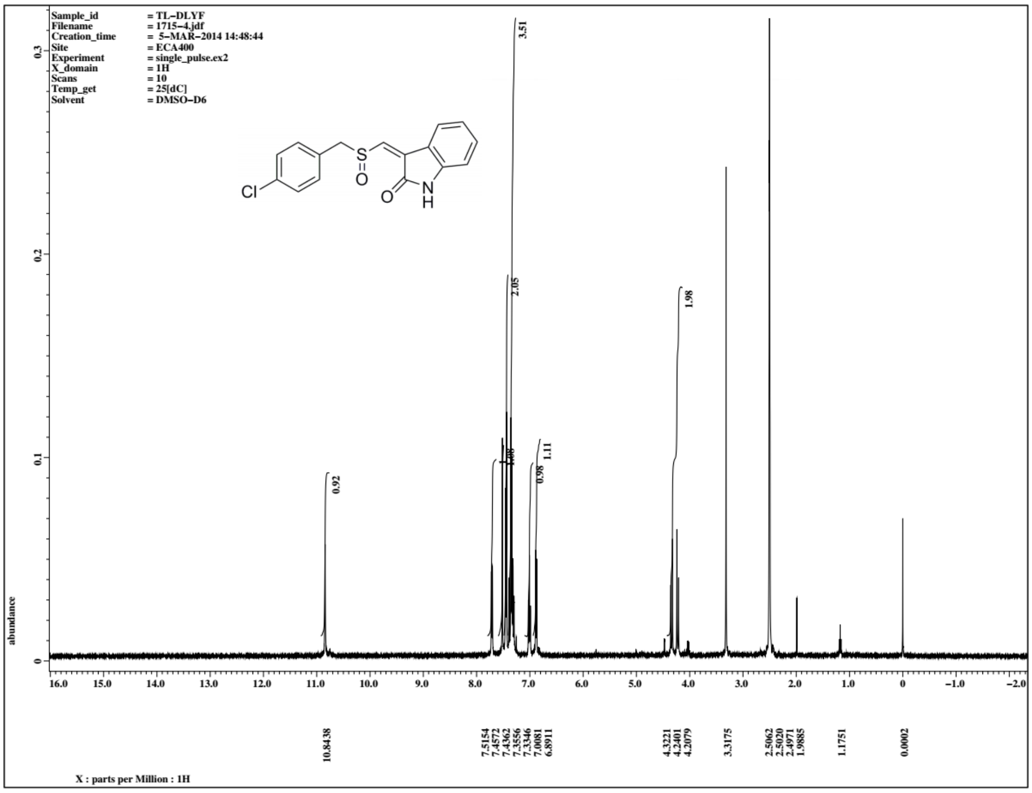

[1]C-NMR of Compound 6g:

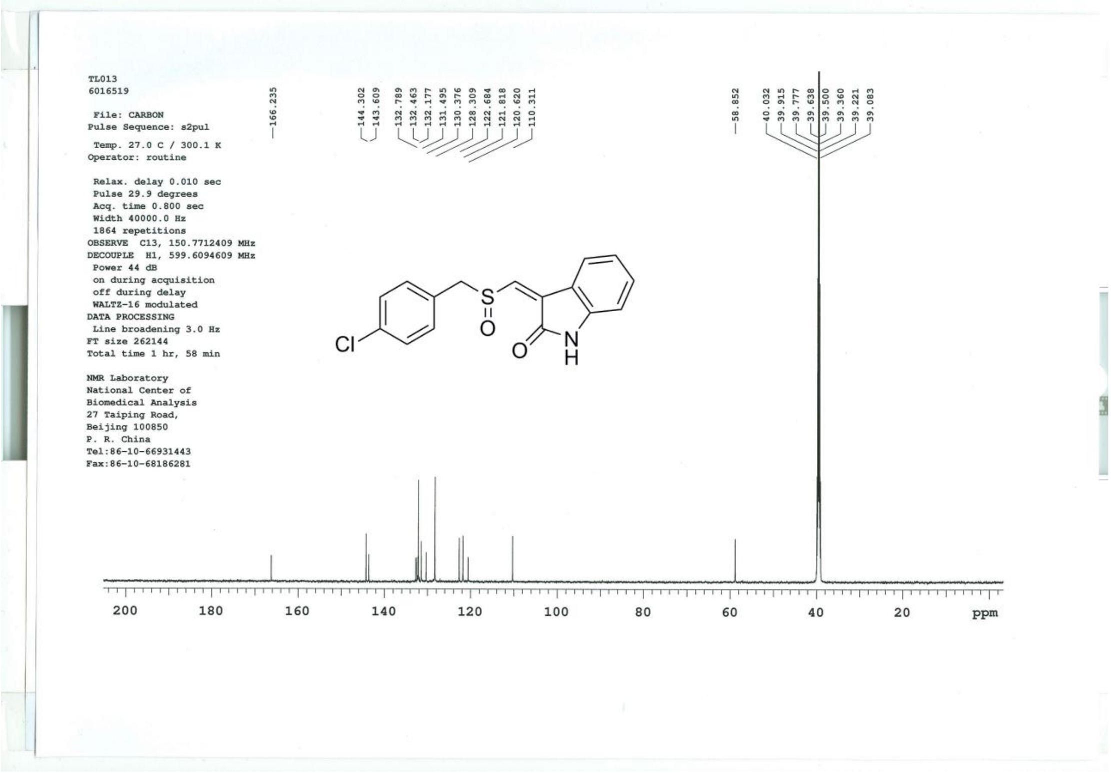

1H-NMR of Compound 6h:

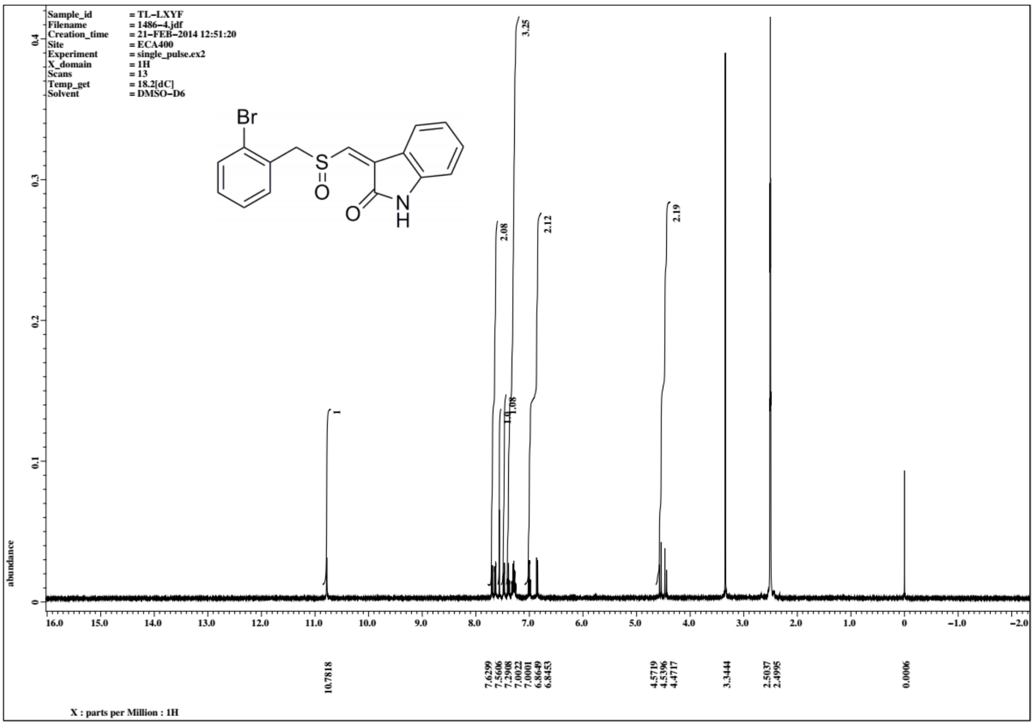

1C-NMR of Compound 6h:

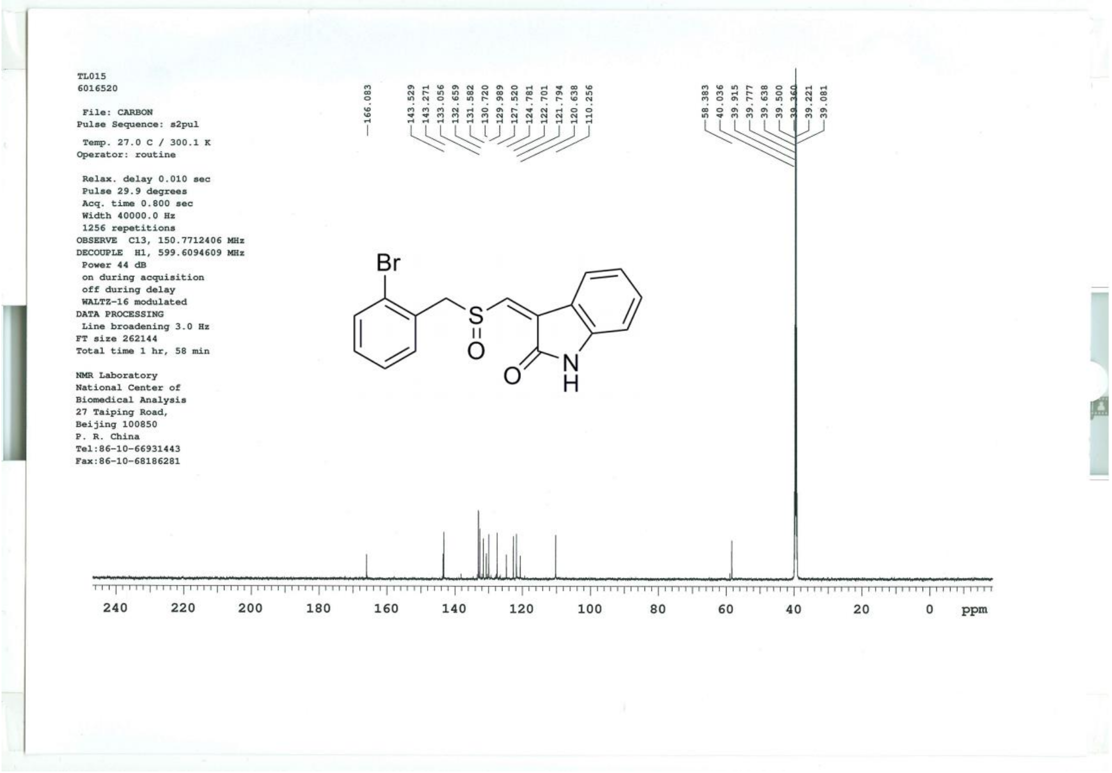

^{1}H-NMR of Compound 6i:

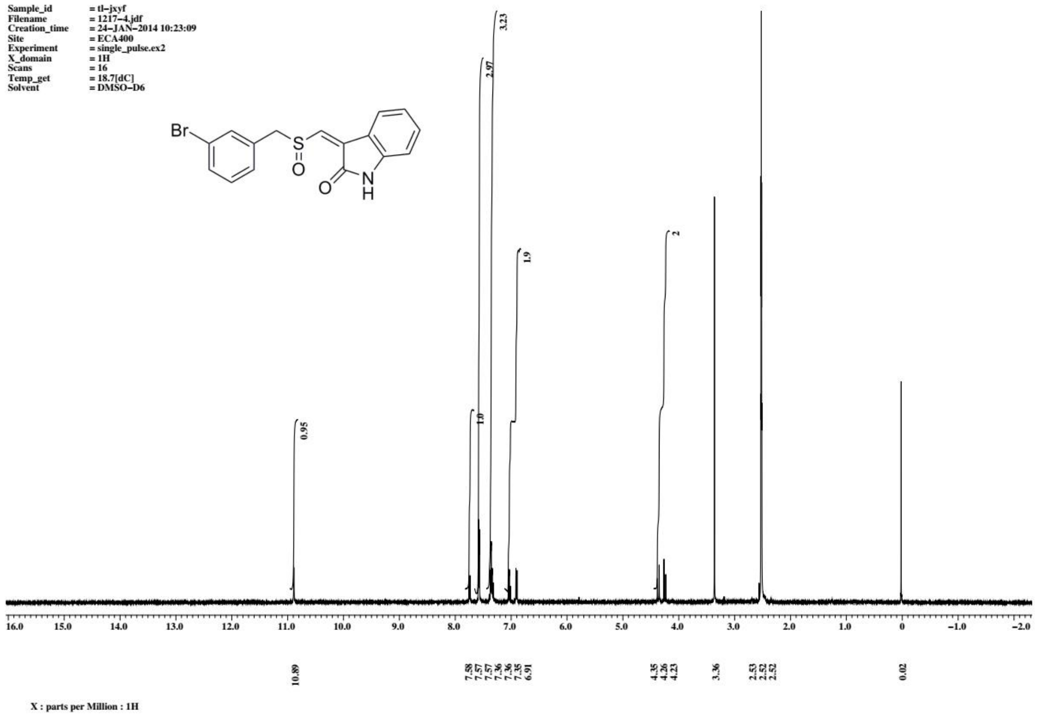

[1]C-NMR of Compound 6i:

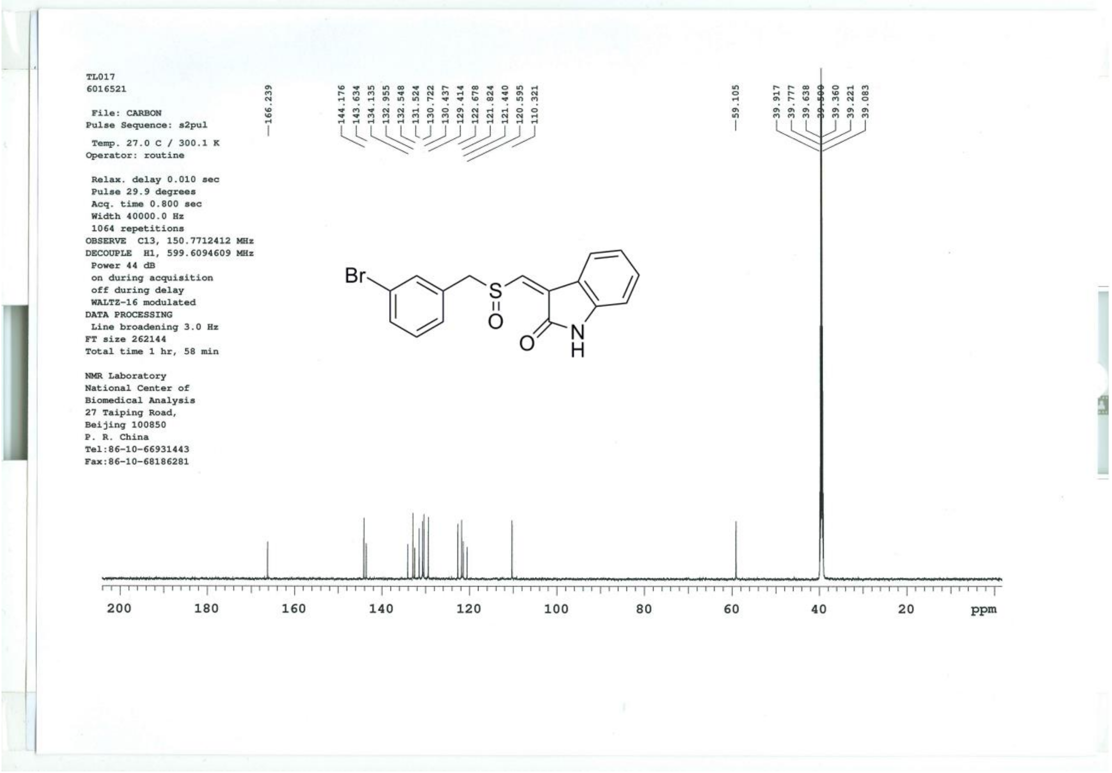

^{1}H-NMR of Compound 6j:

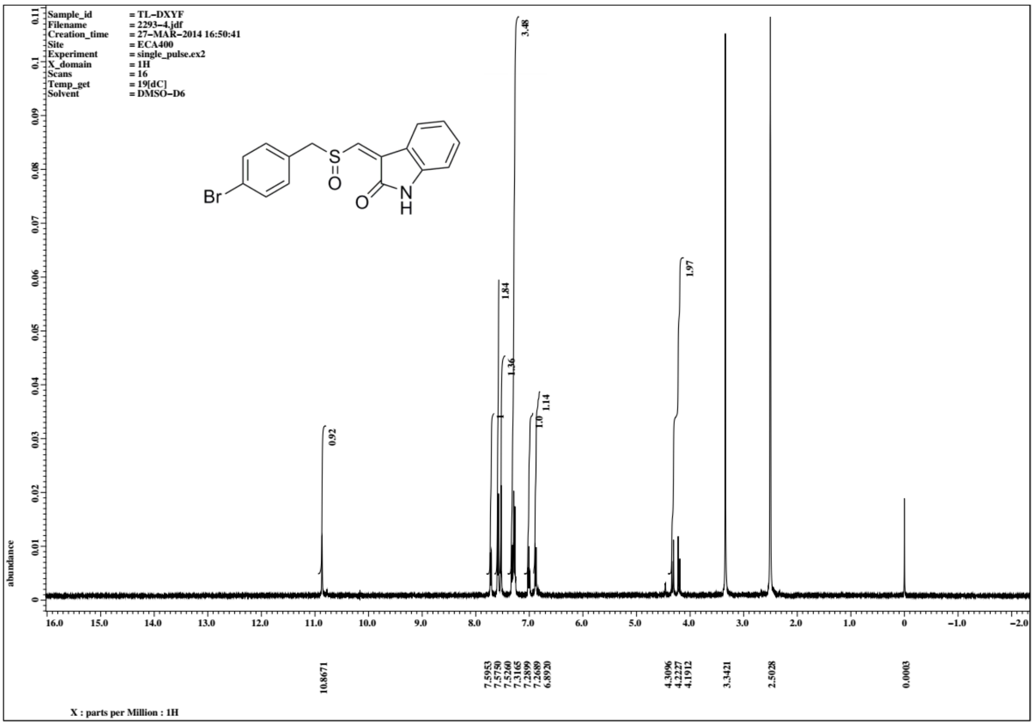

[1]C-NMR of Compound 6j:

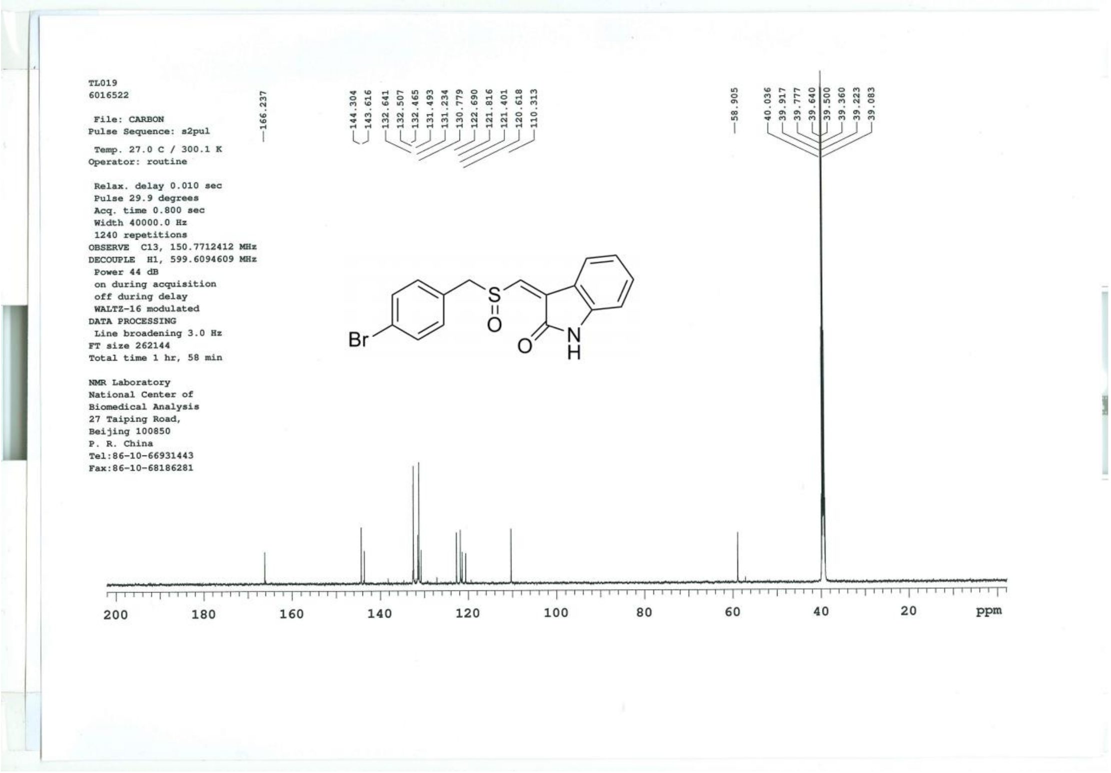

^{1}H-NMR of Compound 6k:

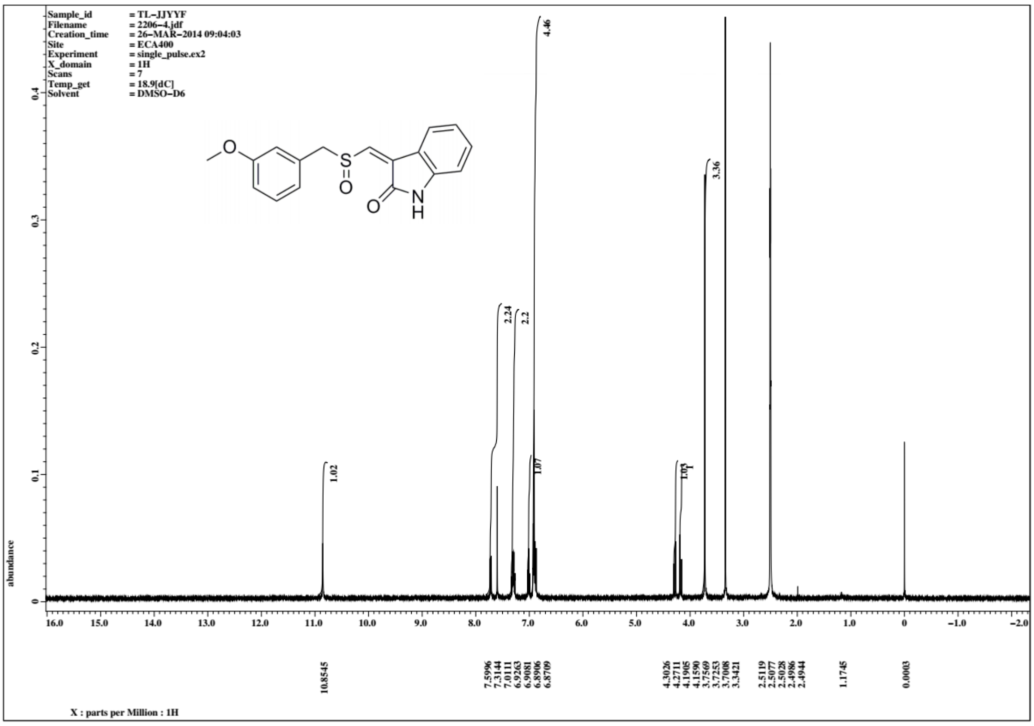

[1]C-NMR of Compound 6k:

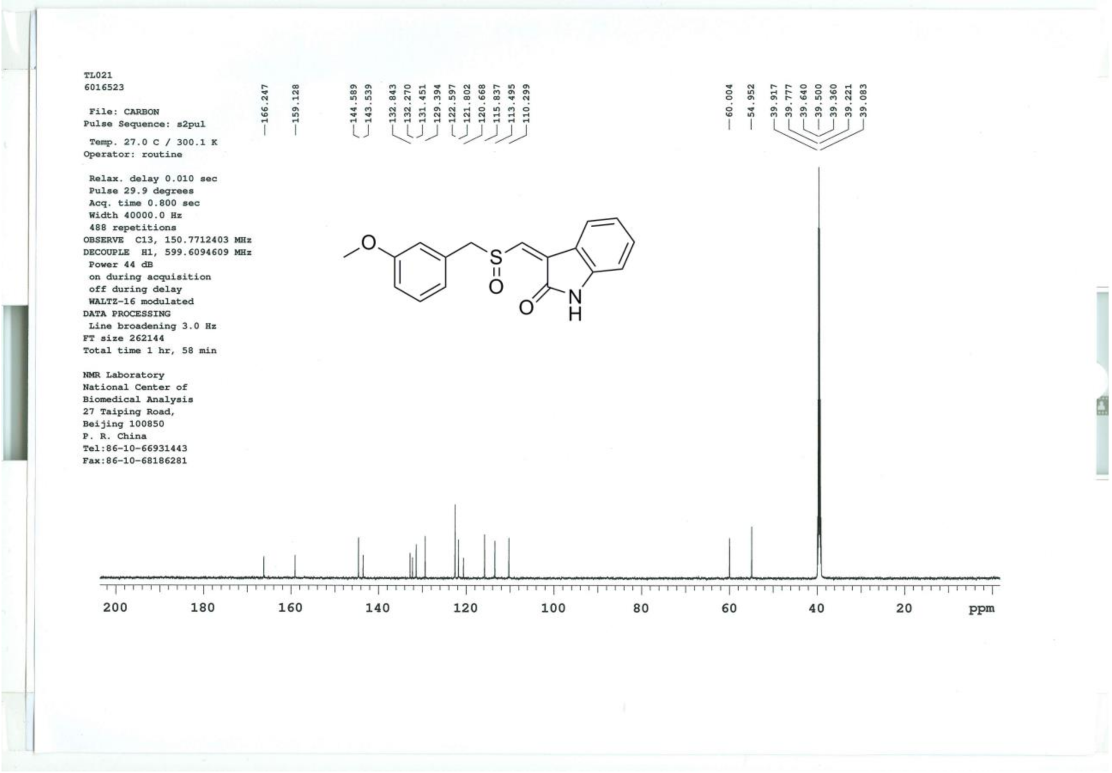

^{1}H-NMR of Compound 6l:

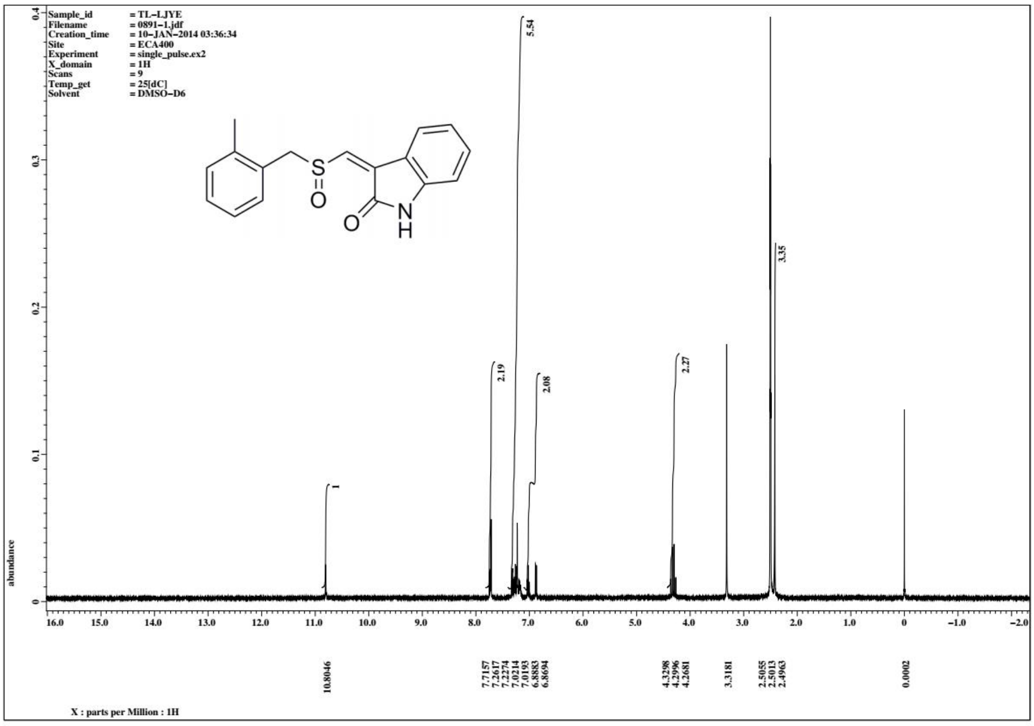

^{1}C-NMR of Compound 6l:

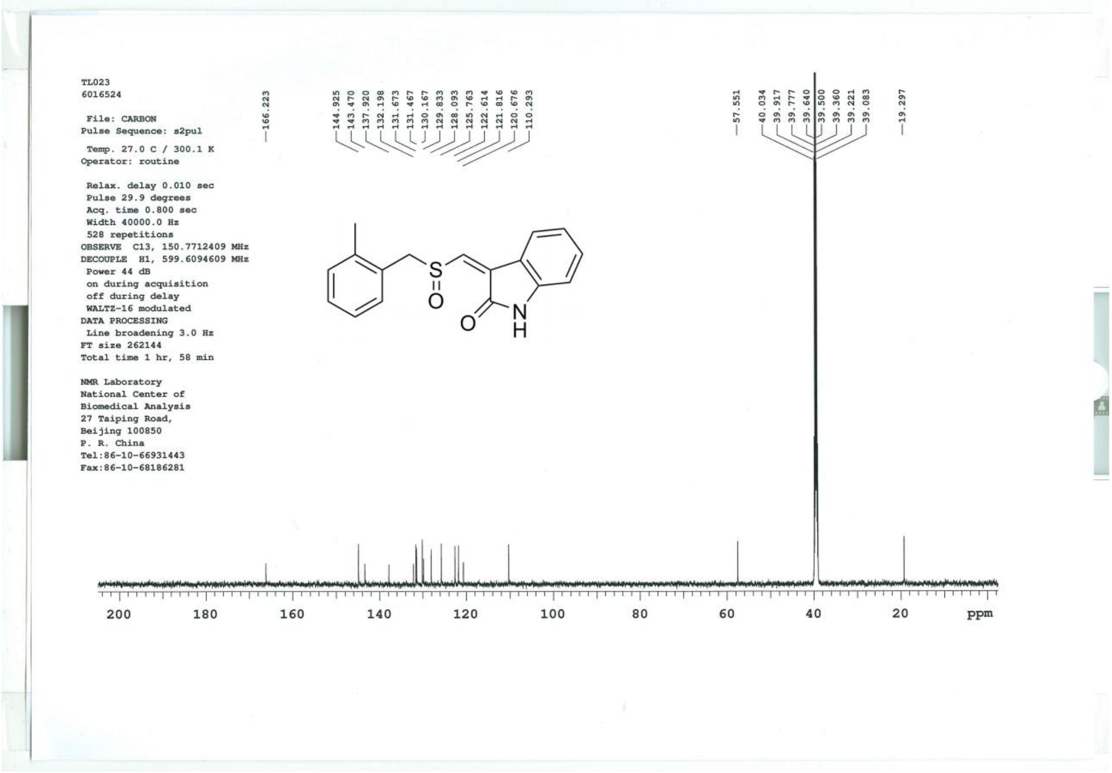

^{1}H-NMR of Compound 6m:

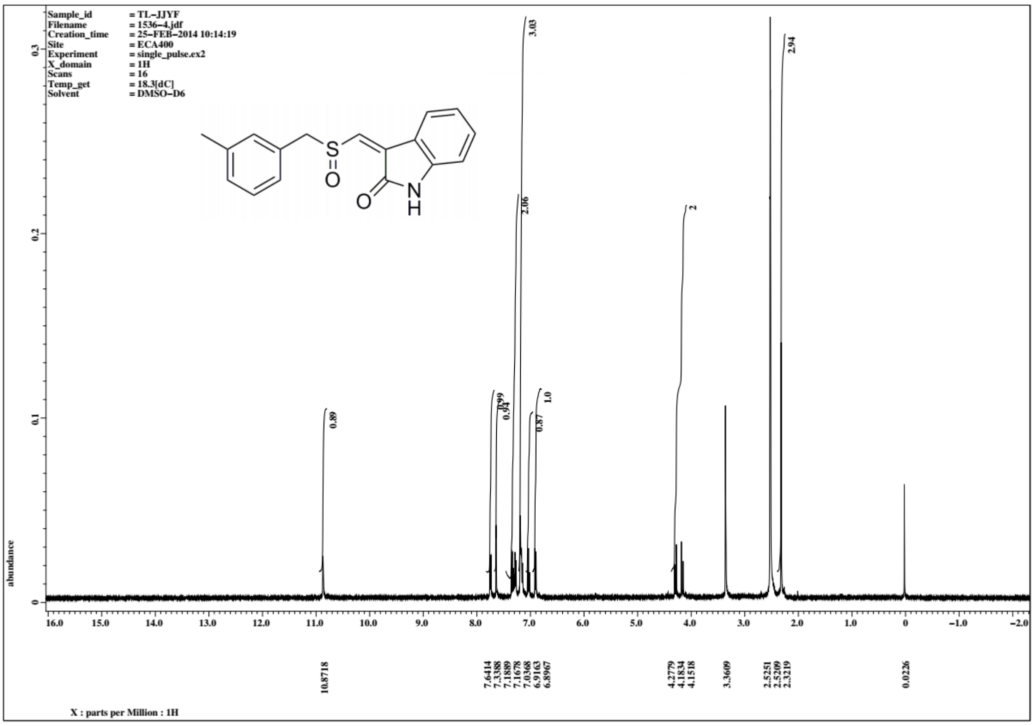

[1]C-NMR of Compound 6m:

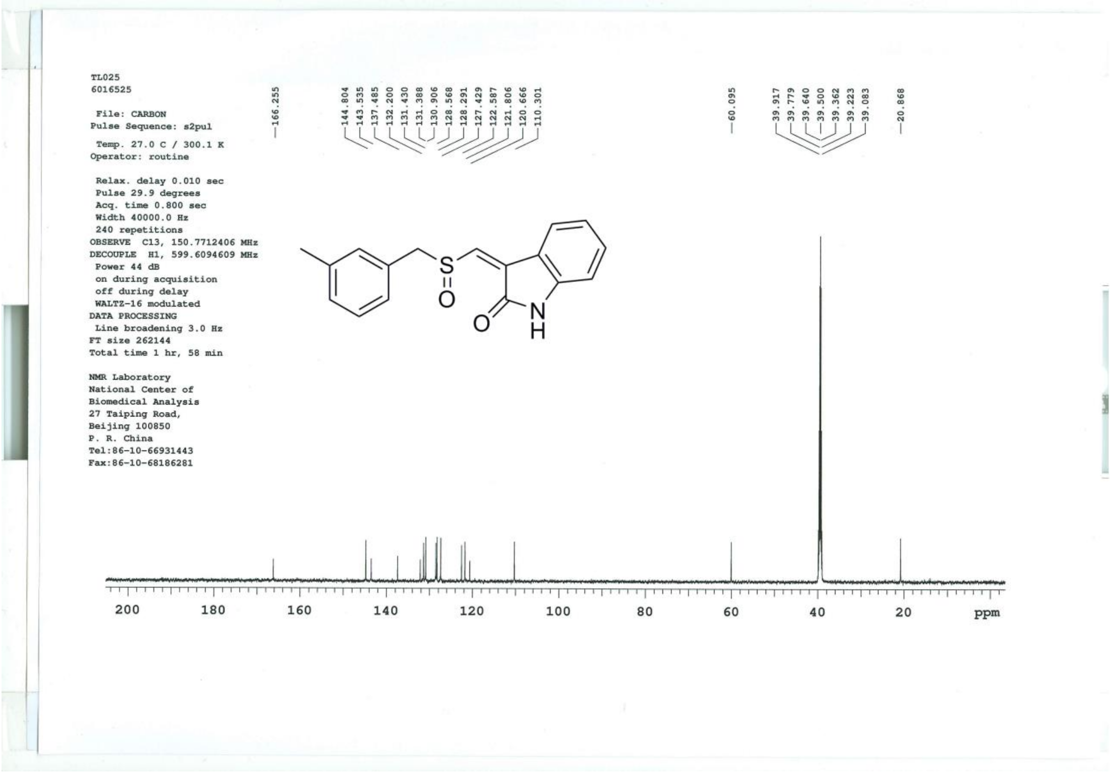

^{1}H-NMR of Compound 6n:

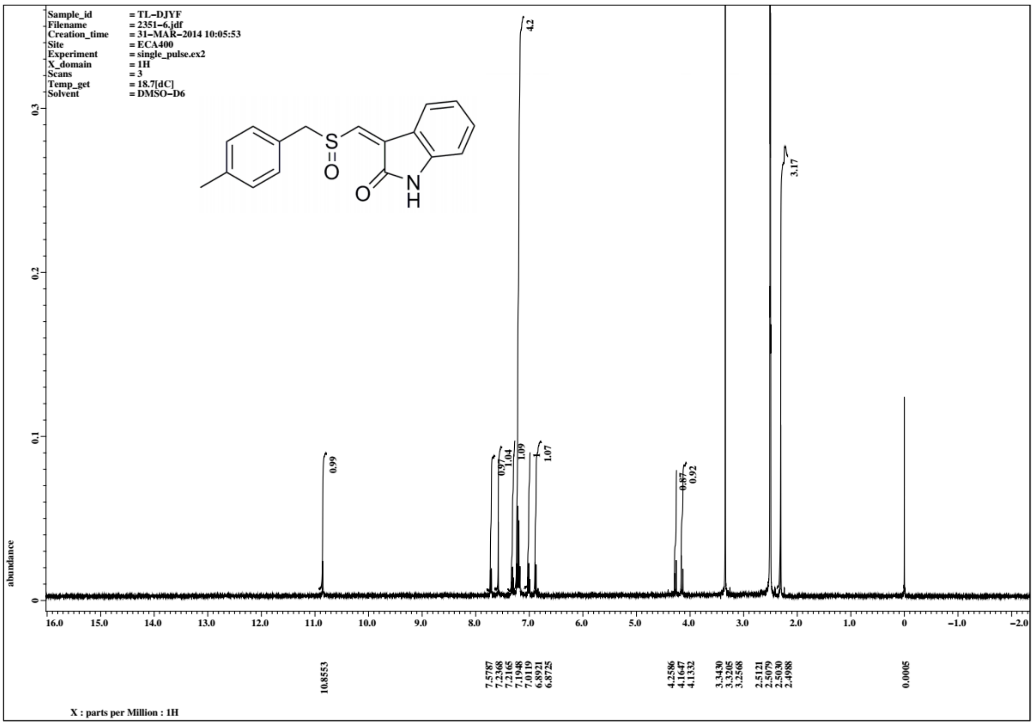

[1]C-NMR of Compound 6n:

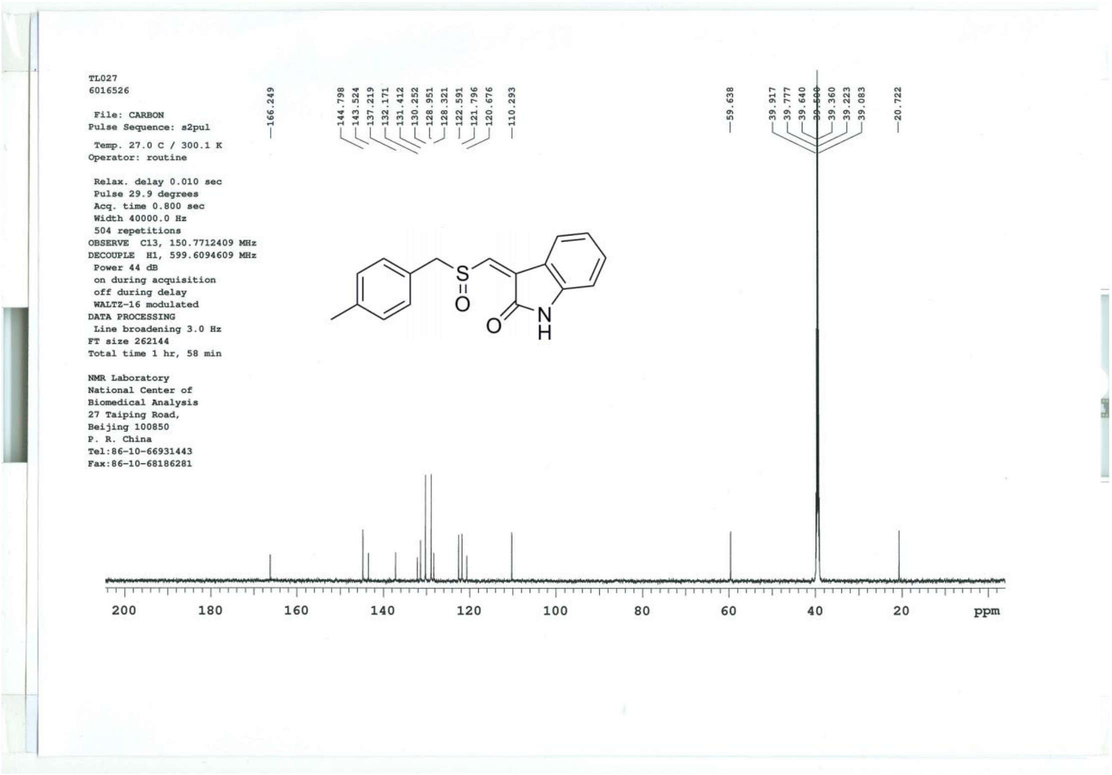

^{1}H-NMR of Compound 6o:

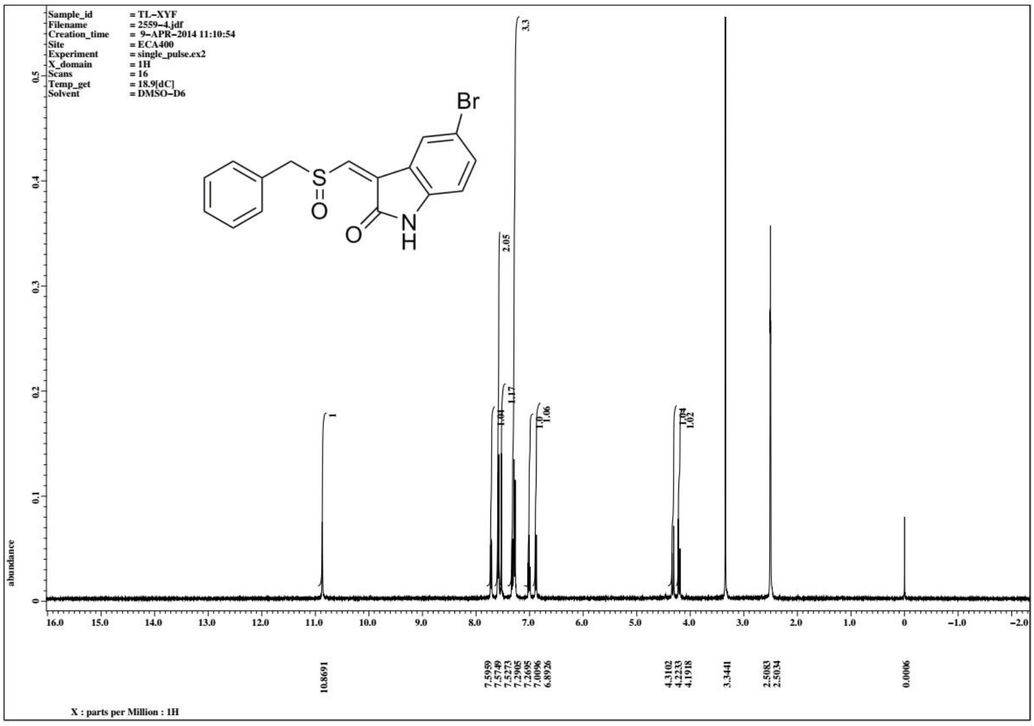

^{1}C-NMR of Compound 6o:

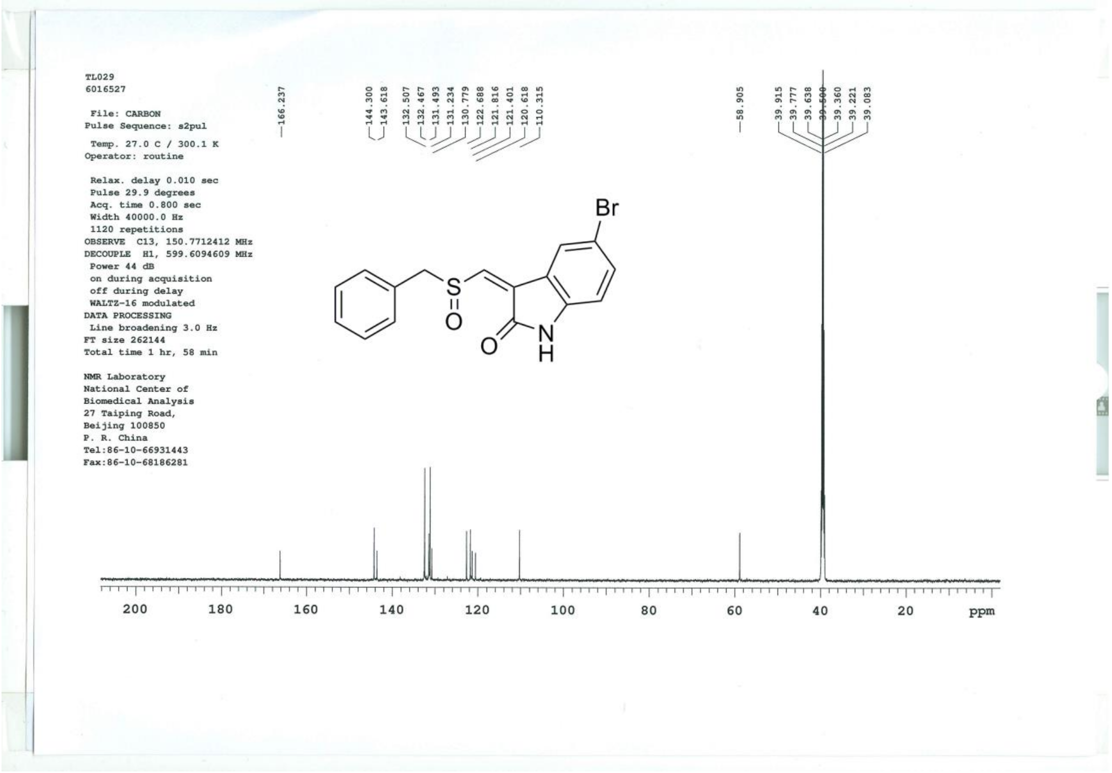

^{1}H-NMR of Compound 6p:

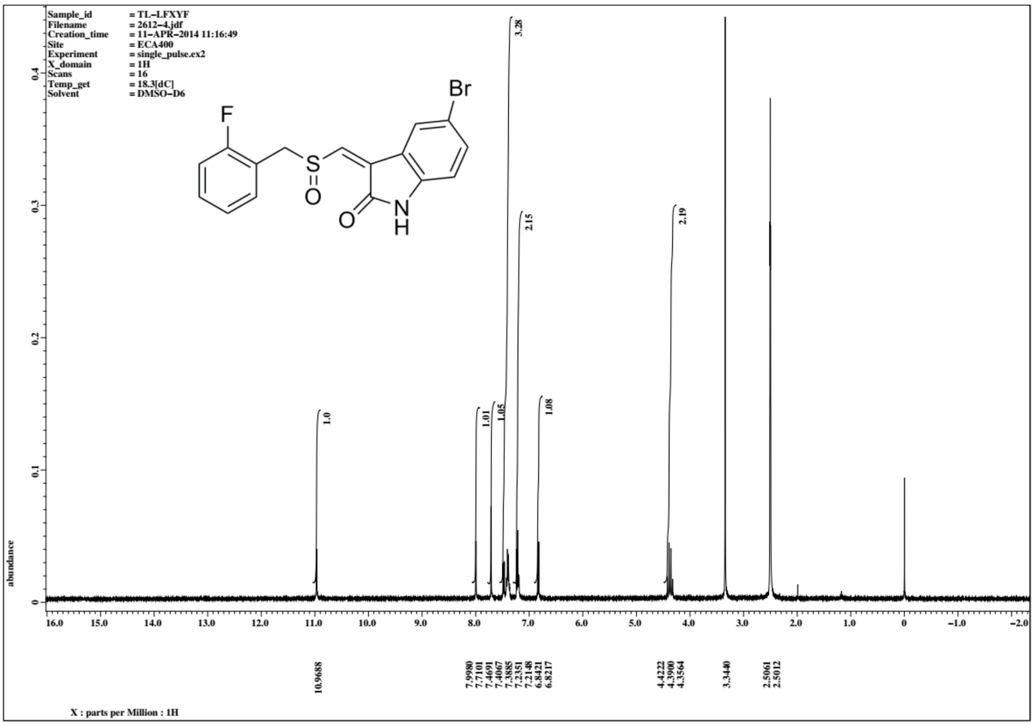

[1]C-NMR of Compound 6p:

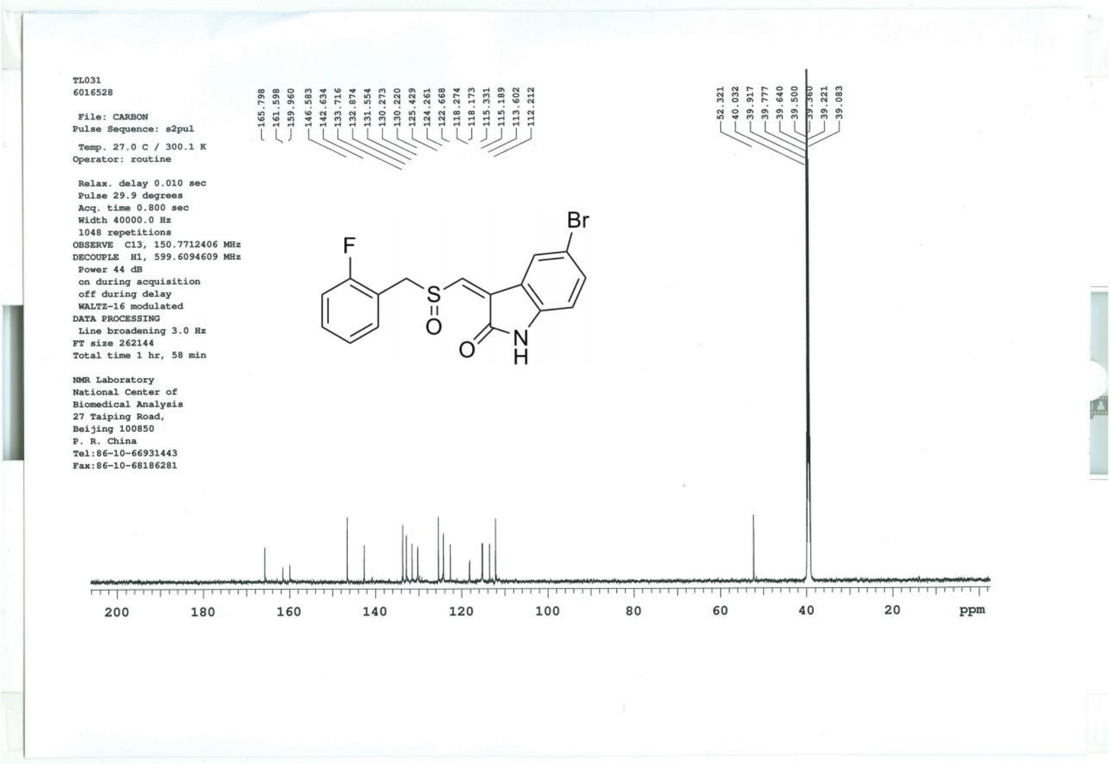

Supplement: Supplementary file 1 [file molecules-22-01979-s001.pdf]
